# A Review of Probiotic Interventions for Necrotizing Enterocolitis and Sepsis in Preterm Infants

**DOI:** 10.3390/ijms27083602

**Published:** 2026-04-17

**Authors:** Angel Yun-Kuan Thye, Hui Xuan Lim, Yatinesh Kumari, Loh Teng-Hern Tan, Vengadesh Letchumanan, Priyia Pusparajah, Kok-Gan Chan, Learn-Han Lee, Jodi Woan-Fei Law

**Affiliations:** 1 Next-Generation Precision Medicine and Therapeutics Research Group (NMeT), Jeffrey Cheah School of Medicine and Health Sciences, Monash University Malaysia, Bandar Sunway 47500, Selangor Darul Ehsan, Malaysia; angel.thye1@monash.edu (A.Y.-K.T.); loh-teng-hern.tan@nottingham.edu.cn (L.T.-H.T.); kokgan@um.edu.my (K.-G.C.); 2 Pathogen Resistome Virulome and Diagnostic Research Group (PathRiD), Jeffrey Cheah School of Medicine and Health Sciences, Monash University Malaysia, Bandar Sunway 47500, Selangor Darul Ehsan, Malaysia; vengadesh.letchumanan1@monash.edu; 3 Department of Biomedical Sciences, Sir Jeffrey Cheah Sunway Medical School, Faculty of Medical and Life Sciences, Sunway University, Sunway City 47500, Selangor Darul Ehsan, Malaysia; huixuanl@sunway.edu.my; 4 Sunway Microbiome Centre, Faculty of Medical and Life Sciences, Sunway University, Sunway City 47500, Selangor Darul Ehsan, Malaysia; 5 Neurological Disorder and Aging (NDA) Research Group, Neuroscience Research Strength (NRS), Jeffrey Cheah School of Medicine and Health Sciences, Monash University Malaysia, Bandar Sunway 47500, Selangor Darul Ehsan, Malaysia; yatinesh.kumari@monash.edu; 6 Microbiome Research Group, Research Centre for Life Science and Healthcare, Nottingham Ningbo China Beacons of Excellence Research and Innovation Institute (CBI), University of Nottingham Ningbo China, Ningbo 315000, China; 7 Faculty of Medicine, Nursing and Health Sciences, Monash University, Clayton, Melbourne, VIC 3800, Australia; priyia.pusparajah@monash.edu; 8 Division of Microbiology and Molecular Genetics, Institute of Biological Sciences, Faculty of Science, University of Malaya, Kuala Lumpur 50603, Wilayah Persekutuan Kuala Lumpur, Malaysia

**Keywords:** necrotizing enterocolitis (NEC), sepsis, preterm infants, gut microbiome, probiotic

## Abstract

Necrotizing enterocolitis (NEC) and sepsis/late-onset sepsis (LOS) are significant contributors to preterm infant morbidity and mortality, with prematurity and low birth weight representing major risk factors for these interconnected conditions. Although the pathogenesis of NEC and LOS is not fully understood, there is a clear association with an immature intestinal mucosal barrier, which may enable bacterial invasion and translocation, resulting in an inflammatory cascade. Increasing recognition of the gut microbiome as a marker for health and disease has driven interest in probiotics, particularly *Bifidobacterium* spp. and *Lactobacillus* spp., as potential adjunctive agents for the prevention and management of NEC and LOS in preterm infants, which is the area of focus of this review. The focus of this paper was to analyze clinical studies using different probiotic strains, and compare single-strain versus multi-strain probiotic formulations. Several studies support that probiotic supplementation in preterm infants has the potential to decrease NEC incidence and, to a lesser extent, sepsis/LOS. Nonetheless, inconsistent results due to strain differences and clinical heterogeneity limit the widespread adoption of this mode of therapy, as do safety concerns in this vulnerable population. Further high-quality standardized studies are necessary to establish consistent guidelines for probiotic use in preterm infants.

## 1. Introduction

Preterm birth is defined by the World Health Organization (WHO) as births < 37 weeks of gestation or <259 days from the first date of a woman’s last menstrual period [1]. Preterm birth can be divided into sub-categories based on gestational age, namely: moderate–late preterm (32–37 weeks); very preterm (28–32 weeks); and extremely preterm (<28 weeks) [2]. Statistics indicate that 1 in 10 babies are born prematurely, with approximately 1 million child deaths per year as a result of preterm birth complications [2]. There are a wide range of etiologies for preterm birth, but they can generally be classified as those that result in spontaneous preterm labor (typically associated with inflammation and/or infection) and those that are medically induced as a result of maternal or fetal compromise (typically associated with compromised circulation to the fetus) [1,3,4]. All of these causes create an environment which is likely to result in damage to the fragile developing organs, exacerbating the precarious physiology of the babies born with immature organs not yet intended to be at a stage to support independent existence outside the womb.

Despite great improvements in antenatal and neonatal care leading to an increasing survival rate in recent decades, preterm birth is still a major cause of infant mortality and morbidity [5]. These infants often experience long-term damage to various organ systems as a result of factors inherently related to preterm birth, namely: (1) factors precipitating preterm birth (for example, inflammation and infection); (2) physiologic instability of immature organs to transition to and sustain extrauterine life; (3) inadequate synthesis of endogenous protective factors (for example, cortisol and thyroxine); and (4) limited therapeutic modalities available due to the lack of safety data on these treatments [6]. These then result in the myriad complications of prematurity spanning nearly every organ system—bronchopulmonary dysplasia, intraventricular hemorrhage, retinopathy of prematurity, necrotizing enterocolitis, sepsis, and death [6,7,8,9]. In spite of improved survival rates of premature infants, they often have long-term morbidities including cerebral palsy; intellectual disabilities; learning disabilities; and behavioral, social, and emotional issues that often persist into adulthood [8,10].

NEC and LOS are major morbidities in preterm infants [11,12,13,14,15]. NEC affects between 2 and 7% of all preterm births, with 90% of cases occurring in newborns less than 32 weeks of gestation [11]. A recent multicenter cohort study evaluating 25,821 infants from 2005 to 2017 showed an incidence of 8.8% that remained stable over the study period, though there was improvement in mortality from 36.7% between 2005 and 2008 to 26.6% between 2015 and 2017 [12]. Although the mortality reduction is encouraging, the high incidence of mortality still indicates a need for more effective management strategies. NEC is among the most common gastrointestinal emergencies that impact preterm infants who have survived the early neonatal period [16]. The hallmark of NEC is gastrointestinal dysfunction that can progress to pneumatosis intestinalis, pneumoperitoneum, systemic shock, and, in severe cases, rapid death [17]. On the other hand, sepsis is marked by overactivation of both pro-inflammatory and anti-inflammatory pathways; stimulation of the coagulation cascade and complement system; sepsis-induced neutropenia and thrombocytopenia; and biochemical imbalances that lead to an oxidant state linked with decreased levels of antioxidant (for example, glutathione) in both plasma and tissues [18]. LOS is defined as sepsis occurring after 3 days of age [19,20]. In preterm infants, it can progress quickly from sepsis to septic shock, which is associated with high mortality rates [14]. The onset of both NEC and sepsis is rapidly progressive, involving multiple organ systems and an intense inflammatory response, and is reflected by survivors with poor growth and developmental delays [21]. Studies have shown substantial evidence that gut dysbiosis was implicated in both NEC and sepsis in preterm infants [22,23].

Importantly, NEC and sepsis are interconnected, perhaps unsurprisingly so given that they share common risk factors of prematurity and low birth weight, with gestational age and birth weight showing an inverse relationship with incidence and mortality rate [16,24]. Recurrent sepsis is one of the significant long-term complications of NEC, and NEC is one of the complications of prematurity associated with an increased rate of LOS [13,25]. NEC arises from bacterial translocation that triggers an inflammatory cascade. This response leads to the perpetuation of a vicious cycle of tissue injury and inflammation, resulting in extensive microbial invasion of the intestinal wall, intestinal necrosis or perforation, and systemic sepsis [25]. There are parallels with LOS survivors who are at risk of developing bronchopulmonary dysplasia, neurodevelopmental impairment, prolonged hospitalization, and NEC [15]. In preterm infants, treatment with antibiotics, delayed enteral feeding, and impaired colonization of healthy intestinal bacteria are common and play a part in increasing the risk of NEC and sepsis (a complication of as well as a risk factor for NEC) [15,26]. The exact causes of NEC and LOS are not clearly delineated, making early and accurate diagnoses more challenging, and particularly making it challenging to institute appropriate treatment, with the challenge being amplified by the fact that current therapeutic modalities often had limited efficacy [16,19,27,28].

Premature infants are especially vulnerable to developing NEC due to the underdevelopment of crucial physiological functions, including circulatory regulation, digestive ability, gastrointestinal mobility, intestinal barrier integrity, and immune defense mechanisms. Other potential contributing factors may include colonization by pathogenic bacteria, feeding formula milk, and hypoxic–ischemic injury [16,17,29,30,31]. Preterm infants are also vulnerable to nosocomial infections due to impaired host immune defense mechanisms; decreased skin barrier function; limited amounts of protective endogenous flora on skin and mucosal surfaces at the time of birth; frequent broad-spectrum antibiotic exposure; and invasive devices and procedures [26,32,33]. Importantly, alterations in the gut microbiome and an immature mucosal barrier could affect immune response, metabolic function, and tight junction integrity, which subsequently increases the risk of preterm infants to LOS [23,34,35].

Growing evidence highlights the role of the gut microbiome in health and diseases [36,37,38,39,40,41,42,43], with increasing recognition of its importance in preterm infants [23,44]. Accordingly, probiotics are being investigated as a potential adjuvant strategy for the prevention and/or treatment of NEC and sepsis/LOS in preterm infants.

## 2. Necrotizing Enterocolitis (NEC) and Sepsis

NEC is the most common and lethal gastrointestinal disease affecting preterm infants, with a mortality rate between 20 and 30% [45]. Survivors often have a high risk of permanent impairment of gut function and neurodevelopmental delays [45]. A systematic review and meta-analysis by Alsaied et al. [46], reported that 7 out of 100 of all very low birth weight infants in the NICU are likely to develop NEC; however, considerable heterogeneity existed in the estimates across studies. An important point to note is that NEC is one of the conditions often linked with late-onset bacteremia [18]. Furthermore, in infants with clinically evident NEC, concurrent bloodstream infections primarily caused by Gram-negative bacteria were identified in 40–60% of cases [18,47].

The clinical presentation of NEC in neonates includes feeding intolerance, delayed gastric emptying, abdominal distension or tenderness (or both), occult or gross blood in the stool, apnea, lethargy, poor perfusion, and respiratory distress. Early signs of NEC in a preterm neonate have many features in common with sepsis which makes an accurate diagnosis challenging, though the initial treatment of both has many elements in common [16]. Immaturity of the intestinal mucosal barrier function appears to have a definite role in the pathogenesis of NEC, possibly facilitating invasion and translocation of pathogenic bacteria [21], with the immature intestinal host defense system possibly resulting in increased adhesion of pathogenic bacterial species [48]. It has been hypothesized that NEC starts with a breach in the intestinal mucosal barrier, triggering bacterial translocation across the epithelium and exacerbation of the inflammatory cascade, leading to the clinical signs of NEC [48,49,50,51]. One study reported that the hallmark of NEC is an excessive inflammatory response of the immature gut, resulting from the developmental immaturity in innate immune response genes [50]. Nanthakumar et al. [49] showed that, compared to the mature enterocytes of older children, the premature human enterocyte possesses an increased IL-8 response to inflammatory stimuli, which may partly explain the pathophysiology of NEC. Overall, the current knowledge suggests that insults to an immature mucosal barrier and intestinal host defense system, followed by bacterial translocation, lead to an exacerbation of the inflammatory cascade in the premature gut resulting in the clinical picture of NEC.

Antibiotic exposure prior to NEC is an important clinical determinant of NEC incidence, as this affects the development of preterm infants’ gut microbiomes [22]. NEC has been associated with antibiotic exposure, which supports the idea of intestinal bacteria involvement in its pathogenesis [22]. A systematic review and meta-analysis by Pammi et al. [22] reported intestinal dysbiosis preceding the onset of NEC in preterm infants, characterized by a decreased relative abundance of *Firmicutes* and *Bacteroidetes* and an increased relative abundance of *Proteobacteria*; notably, this microbial signature overlaps with dysbiosis observed following antibiotic exposure (increased *Proteobacteria* and decreased *Firmicutes*) [22]. Antibiotic usage is associated with an increased abundance of *Proteobacteria*, which is associated with a higher incidence of NEC. Hence, this supports an association between prior antibiotic use and an increased NEC incidence [22]. Additionally, a study involving a twin pair discordant for NEC by Stewart et al. [52] found obvious changes in the composition of intestinal flora attributable to antibiotic exposure and associated with the development of NEC, with effects that reduced diversity and increased *Escherichia* sp. dominance preceding NEC, followed by an increased abundance of other *Enterobacteriaceae* and a reduced abundance of *Escherichia* sp. after antibiotic treatment. Our review of the literature indicated that no single causative agent has been identified as a cause of NEC; instead, the data seems to be moving toward a focus on the populations of bacteria in the intestine of preterm infants that disrupt or promote processes associated with intestinal epithelial barrier maintenance [45]. These findings suggest a strong association between gut dysbiosis and the pathogenesis of NEC.

Clinically, the severity of NEC is graded using the modified Bell’s criteria. Thus, staging utilizes a combination of historical, clinical, and radiographic data and includes three main stages: stage I (suspected), stage II (definite), and stage III (advanced). Bell’s criteria have been widely used as a classification system for diagnosing and assessing the severity of NEC. In1978, Bell et al. [53] proposed the clinical staging criteria for infants with NEC. This was, however, modified by Walsh and Kleigman et al. [54] in 1986, and was termed as the modified Bell’s criteria. The original Bell’s criteria were based upon historical, clinical, and radiographic data, and were categorized into three main stages: stage I (suspected), stage II (definite), and stage III (advanced) [53]. At least one or more histological factors will be present during all stages. During stage I, clinical signs include poor feeding; mild abdominal distension; emesis; increasing pregavage residuals; occult blood may be present in the stool (no fissures); lethargy; bradycardia; temperature instability; and apnea. The abdominal radiograph will show distension with mild ileus. During stage II, in addition to the clinical signs in stage I, there will be persistent occult or gross intestinal bleeding; marked abdominal distension; and an abdominal radiograph showing significant intestinal distension with ileus; small bowel separation; unchanging or fixed bowel loops; pneumatosis intestinalis; and portal vein gas. During stage III, the deterioration of vital signs and evidence of septic shock or marked gastrointestinal hemorrhage are present, and a radiograph may show pneumoperitoneum in addition to those mentioned in stage II [53]. Later, in the modified Bell’s criteria, more detailed sub-stages (stages IA, IB, IIA, IIB, IIIA, IIIB) were added [54]. Nonetheless, there are some recent studies that presented NEC assessment tools that are based on a combination of clinical signs, laboratory findings, radiological findings, and, in some cases, surgical or postmortem confirmation [55,56].

Neonatal sepsis, on the other hand, is defined as an infection in the first 28 days of life, or up to 4 weeks after the expected due date for preterm infants [57]. It is a life-threatening condition resulting from systemic infections that trigger a chain of overwhelming inflammatory immune responses [18]. Neonatal sepsis can be a consequence of infections with bacterial, viral, or fungal microorganisms [58]. Infant risk factors of neonatal sepsis include prematurity or low birth weight (with 3–10 times higher rates of infection than full-term normal birth weight infants) [58]. Neonatal sepsis can be divided based on the age of onset and the timing of episodes into early-onset or late-onset. Early-onset infections are acquired before and during delivery, and manifest in the first 72 h of life (up to 7 days), while late-onset infections are acquired after delivery and peak in the second to third week of postnatal life [57,58]. This classification aids in directing antibiotic treatment, as it implies variations in the presumed mode of transmission and predominant organism involved [28]. Group B *Streptococcus* and *E.coli* are the most frequently implicated organisms in early-onset sepsis (EOS), whereas coagulase-negative staphylococci, *Staphylococcus aureus*, and Gram-negative rods such as *Escherichia coli*, *Klebsiella* spp., *Enterobacter* spp. and *Serratia* spp. are more commonly associated with LOS [59]. EOS is acquired via vertical transmission from mother to infant prior to or during delivery [27,59]. In contrast, LOS is predominantly acquired from the postnatal environment, including exposure to invasive devices, prolonged parenteral feeding, and contact from caregivers or healthcare workers [15,60], and is therefore more likely to be amenable to prophylactic interventions.

While both EOS and LOS are of clinical significance, focus has been placed on LOS given its high incidence, associated mortality, treatment complexity, and strong associations with preterm infants’ immature immunity, altered gut microbiota, and prolonged hospitalizations [18,61,62,63,64,65,66]. Neonatal LOS has an incidence of 15–27.6% in very low birth weight infants [14], with incidence rates in preterm infants varying between 20 and 38% in the first 120 days of life [15]. LOS occurs after 72 h of birth and is therefore mainly acquired from hospitals [14], though it can be acquired horizontally from the community or hospital environment, or from maternal vertical transmission [27]. The risk factors of LOS include prematurity, a low birth weight, invasive procedures, prolonged indwelling catheter use, ventilator-associated pneumonia, prolonged usage of antibiotics, and prolonged hospitalization [14,28]. The incidence of LOS shows geographic variation [14]. LOS is associated with an 18% mortality rate and extended hospital stay [13,20], with survivors also at an increased risk of neurodevelopmental impairment and developing NEC and bronchopulmonary dysplasia [15].

The conventional blood culture is the gold standard for diagnosing LOS and identifying the pathogen [67,68], which are necessary for the accurate diagnosis and adjustment of empiric antibiotic treatment [69]. However, the results from blood cultures are not available immediately, and pathogens are detected only in 25% of cases [67]; furthermore, with regard to preterm infants, blood cultures have a sensitivity of 10–20% as a result of low sample volumes, low-level bacteremia, and previous antibiotic treatments [23]. Nonetheless, it is important to note that despite having a negative blood culture, sepsis cannot always be ruled out. On the contrary, the detection of bacteria in a blood culture may reflect contamination or asymptomatic bacteremia [28]. This has led to the investigation of additional diagnostic methods, including non-culture-based techniques, immune biomarkers, and the measurement of serine protease inhibitors such as inter α inhibitor proteins (IAIP) which may improve early detection of neonatal sepsis [28]. That being said, the presenting symptoms of LOS may be subtle and non-specific; hence, an early and correct diagnosis is challenging. The most important clinical signs suggestive of being linked with LOS include increased respiratory support, capillary refill, gray skin, and a central venous catheter [19]. It should be noted, however, that a central venous catheter is a risk factor as well as a clinical sign, unless there are local signs of infections, such as erythema, or tracking along the insertion or path of the vein [70,71]. Other clinical signs include apnea, dyspnea, feeding difficulty, irritability, temperature instability, hyper- and hypothermia, and tachycardia [19].

The microorganisms responsible for LOS contribute significantly to determining the overall outcome. Data from the National Institute of Child Health and Human Development Neonatal Research Network centers reported that 70% of infections were a consequence of Gram-positive microorganisms, with coagulase-negative staphylococci responsible for 48% of infections, and that the infection rate of LOS was inversely associated with gestational age and birth weight [13]. Consistent with this, Bizzarro et al. [24] reported that coagulase-negative staphylococci were the most commonly isolated organisms (31%) in LOS, followed by *S. aureus* (17%), *Enterococcus faecalis* (13%), and *E. coli* (11%). However, the greatest sepsis-related mortality was associated with *Pseudomonas aeruginosa* (56%), followed by *E. coli* (20%), *Klebsiella pneumoniae* (13%), and *S. aureus* (12%). The authors also found that the rates of LOS, death, and sepsis-related deaths were inversely proportional to gestational age and body weight [24]. Some other microorganisms involved in neonatal LOS include group B streptococci, *Enterococcus* spp., *Klebsiella* spp., *Candida albicans*, and other *Candida* spp. [72,73]. All in all, the most commonly isolated pathogens are coagulase-negative staphylococci [24,57,72,73,74]. Given that coagulase-negative staphylococci are a ubiquitous skin commensal, Marchant et al. [57] proposed that indwelling catheters and colonization of the skin are crucial sources of sepsis. This emphasized that the NICU environment can impact the health of preterm infants.

**Figure 1:**
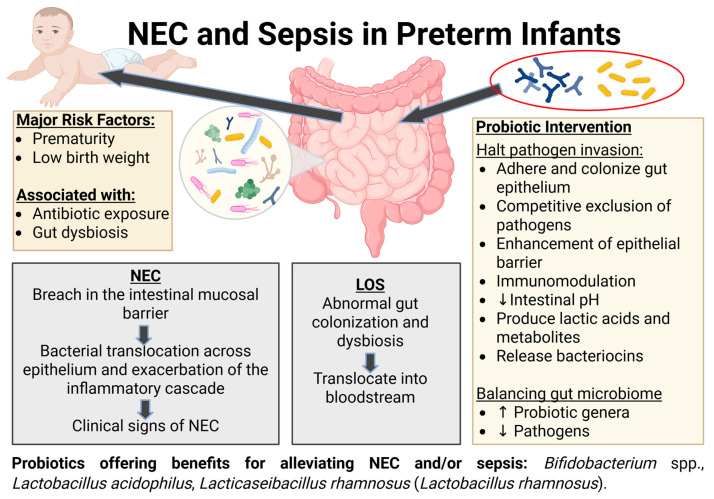
Proposed mechanisms and potential outcomes of probiotic intervention targeting NEC and sepsis among preterm infants. Created in BioRender. Law, J. W. (2026) https://BioRender.com/kej8mh7 (Accessed on 29 March 2026).

Despite the pathogenesis of LOS still being under debate, studies have suggested that pathogenic bacteria enter the bloodstream either from gut-colonizing bacteria or from the environment [27,28]. Similar pathogenic mechanisms are likely implicated in LOS cases when the infecting organism arises from an intestinal reservoir [21], highlighting the connection between NEC and LOS. Interestingly, growing evidence suggests that abnormal gut colonization and dysbiosis are associated with the pathogenesis of LOS, as many organisms implicated are common members of the dysbiotic gut microbiota of preterm infants. For instance, El Manouni El Hassani et al. [64] reported that the causative pathogens, including *E. coli* and *K. pneumoniae*, were detected prior to LOS onset, with pathogen abundance increasing up to 3 days before clinical onset. In addition, de Kroon et al. [75] found that increased fecal *C. albicans* preceded *Candida* LOS onset in preterm infants, implicating the gut as the source of infection. Together, these findings reinforce the hypothesis that LOS in preterm infants often originate from the gut, where dysbiosis and the overgrowth of pathogens precedes microbial translocation into the blood (Figure 1).

## 3. Management Strategies for NEC and LOS in Preterm Infants

Current management for NEC involves bowel rest, bowel decompression (low-intermittent orogastric suction), and broad-spectrum antibiotics (after obtaining cultures) [16,76,77]. Additionally, adjunctive measures/interventions include hematological support (blood product transfusion), pulmonary support (oxygen, ventilation), and cardiovascular support (pressors, volume) as indicated clinically [16,78]. Surgery is indicated in cases of bowel perforation. Approximately 20–40% infants undergo surgery; however, the case fatality rate with surgical intervention is 50% and is highest for the smallest, least mature infants [16]. An ideal strategy would be to prevent the onset of NEC and some potential preventive strategies for NEC in preterm infants are trophic feeding, conservative feeding, and human milk administration [16]. In fact, with regard to human milk, Cortez et al. [79] reported that logistic regression analysis using PMA and PROM as covariates demonstrated a significant reduction in NEC in preterm infants fed exclusively mother’s milk. Dorling et al. [80] investigated different incremental milk-feeding rates in preterm infants and found no significant difference in NEC incidence, indicating that the feeding rate alone may not significantly affect NEC risk. These findings suggest that, while feeding strategy may influence outcomes, exclusive human milk may play a more critical role in the prevention of NEC.

The management of LOS in preterm infants has several parallels with that of NEC and consists mainly of antibiotic therapy and supportive care [81]. Antibiotic therapy consists of empiric treatment (prior confirmation of causative organism) followed by directed treatment (after positive blood culture and exact causative organism determined) [58]. On the other hand, general supportive care includes maintaining a thermoneutral environment; inotropes and steroids; maintenance of adequate tissue perfusion by fluid resuscitation; and optimal oxygenation [81]. In addition, strategies to decrease the incidence of infections involve maternal chemoprophylaxis for the prevention of early-onset group B streptococcal infection; improved central line care with central line bundles; anti-fungal prophylaxis; attention to NICU design and staffing; and hand hygiene procedures [20]. Similar to NEC, feeding breast milk within the first month of life is protective against the development of sepsis in preterm infants [82]. A study by El Manouni et al. [15] found that breast milk feeding was protective against coagulase-negative staphylococcus LOS. This is consistent with Cortez et al. [79], who found preterm infants fed mother’s milk had lower odds of coagulase-negative staphylococcus LOS, which was likely due to a decrease in both the total infections and the total duration of central venous lines.

For both NEC and LOS in preterm infants, probiotics and lactoferrin are potential supplements or adjuvant therapies. The administration of probiotics as an intervention to prevent NEC is biologically feasible, and some studies have previously taken a strong stance in stating that its use is supported by the published clinical evidence with a clear beneficial risk–benefit balance [83]. It should be noted that a recent systematic review has downgraded the certainty of evidence for probiotic supplementation in very preterm or very low birth weight infants, as relatively few of these infants were included in the studies [84]. Similarly, probiotics have been shown to reduce the risk of LOS in preterm infants [85]. Additionally, studies have also shown that lactoferrin alone, or in combination with probiotics, is beneficial in decreasing NEC and/or LOS incidence in preterm infants [86,87,88,89]. However, the evidence for enteral lactoferrin is of low certainty, and the apparent effect size may be inflated due to the inclusion of small studies in systematic reviews [88,90]. Nonetheless, when treating and/or preventing NEC and LOS in preterm infants, the judicious use of antibiotics should be highly prioritized.

## 4. The Effect of Probiotics on NEC and Sepsis in Preterm Infants

Probiotics are widely available in the market as a supplement for improving health outcomes. Probiotics are defined as living microorganisms that confer health benefits to the host when ingested in adequate amounts. They are readily available via functional food and drinks, which makes diet an important factor in human health. Probiotics can modify and reshape the gut microbiome composition by promoting specific beneficial bacteria, leading to an overall better health outcome [91,92].

Probiotics have been proposed as a potential solution to mitigate preterm infants’ gut dysbiosis, allowing for a stable and healthy gut colonization [93], and have emerged as a potential adjunctive agent in the prevention and management of NEC and LOS in preterm infants. According to Beck et al. [94], the primary factor influencing gut microbiome development in preterm infants who received probiotics is the probiotics itself, and it is the main driver in shaping bacterial community both at the taxonomic and functional levels.

The therapeutic potential of probiotics lies in their ability in intestinal immunomodulation, modulating the composition and function of gut microbial communities, suppressing pathogens, and enhancing intestinal barrier integrity [91]. *Bifidobacterium* and *Lactobacillus* are two of the most common and safest human probiotics, as certified by the human Food and Drug Administration (FDA) with the acronym GRAS (Generally Recognized As Safe) and by the European Food Safety Authority (EFSA) with the acronym QPS (Qualified Presumption of Safety) [95].

*Bifidobacterium* spp. are common residents of the human gut and are characterized as non-motile, non-spore-forming, non-gas-producing, saccharolytic Gram-positive, strict anaerobic bacteria. They belong to the family *Bifidobacteriaceae* within the *Actinobacteria* phylum [96,97]. This genus has been shown to be dominant in the gut microbiota of breastfed infants [98]. *Bifidobacterium* spp. have been identified in a study by Turroni et al. [99] as the most abundant bacteria in stool samples of healthy infants who had not received either antibiotics or probiotics, making up 80.6% of the gut bacteria, with *Bifidobacterium longum* and *Bifidobacterium bifidum* representing 56.2% and 10.7% of the species, respectively. The protective mechanisms of *Bifidobacterium* appear to aid in halting pathogen invasion and in immunomodulation of host intestinal epithelium, including the adhesion to gut epithelium followed by colonization; lowering intestinal pH and production of metabolites; release of bacteriocins; enhancement of epithelial barrier; immunomodulatory effects; and competitive exclusion of pathogens [100,101,102,103,104,105,106,107]. On the other hand, *Lactobacillus* spp. are characterized as non-spore-forming Gram-positive rods that belong to the family *Lactobacillaceae* within the *Firmicutes* phylum [108,109]. Most *Lactobacillus* species are facultative anaerobes, and only 20% of *Lactobacillus* species isolated from humans are strict anaerobes. *Lactobacillus* are commonly found in the mouth, gastrointestinal tract, and female genitourinary tract [108]. The number of *Lactobacillus* in the human gut varies based on the age of the host and the location within the gut. The abundance of *Lactobacillus* ranges from 10^5^ CFU (colony-forming unit)/g in the stool of neonates to 10^6^–10^8^ CFU/g in the stool of infants aged > 1 month [109]. They are regarded as protective microbes that function to suppress the growth of pathogenic microorganisms by producing lactic acid and other metabolites [108].

The potential of these probiotic strains in the prevention and/or treatment of NEC and LOS has been demonstrated by various studies, which will be presented as two categories: single-strain probiotics and combinations of multiple-strain probiotics.

### 4.1. The Effect of Single-Strain Probiotics on NEC and Sepsis in Preterm Infants

#### 4.1.1. *Bifidobacterium* spp.

Members of the genus *Bifidobacterium* often dominate the healthy infant gut [110,111]. Bifidobacteria are widely regarded as ideal probiotics for infants owing to their abundance in early-life gut, their positive associations with health, and, importantly, their human milk oligosaccharides (HMOs)-driven symbiotic relationship with humans [110]. Furthermore, the saccharolytic activity creates an anaerobic and acidic gut environment that helps protect against enteropathogenic infections [110]. Some of the common *Bifidobacterium* species used as probiotics in infant studies include *Bifidobacterium longum* subsp. *infantis*; *Bifidobacterium breve*; *B. bifidum*; and *Bifidobacterium lactis*/*Bifidobacterium animalis* subsp. *lactis* [111].

*Bifidobacterium* probiotic strains *B. infantis* (EV C001) [112], *B. breve* [113], and *B. lactis* [114], were associated with decreased NEC in preterm infants across different studies (Table 1). In a nonconcurrent retrospective cohort study by Tobias et al. [112], VLBW infants fed with probiotics *B. infantis* EVC001 (exposed group), at a dose of 8 billion CFU suspended in 0.5 mL of medium-chain triglyceride oil daily via gastric tube before a morning feed, were compared with VLBW infants without the probiotic administration (unexposed group). The findings demonstrated that the cumulative incidence of NEC in infants fed with more than one dose of *B. infantis* EVC001 dropped from 11% to 2.7%—a 73% risk reduction. On the other hand, in terms of the NEC-associated mortality, a drop from 2.7% to 0% was observed when the unexposed group was compared to the exposed group. They found that the administration of *B. infantis* EVC001 as a single-strain probiotic to VLBW infants was associated with significant reductions in the risks of NEC and NEC-related mortality, even in ELBW infants. Also, it may be considered a safe and effective intervention for reducing morbidity and mortality in the NICU [112]. Furthermore, a retrospective cohort study by Patole et al. [113], conducted in Australia, demonstrated that routine probiotic supplementation of *B. breve* M-16V at a dose of 3 × 10^9^ CFU/day to preterm neonates was associated with decreased NEC ≥ stage II and NEC ≥ stage II or all-cause mortality in neonates < 34 weeks. With regard to *B. lactis*, a study in Turkey by Dilli et al. [114] also showed preterm infants administered probiotic *B. lactis*, at a dosage of 5 × 10^9^ CFU with one sachet per day with breast milk or formula milk until discharge or death, for a maximum of 8 weeks, had lower NEC rates (2%) than the placebo (18%) and prebiotic (12%) groups. In fact, the study design included the use of probiotics (*B. lactis*) alone or in combination with prebiotics (inulin), finding an improvement in resistance to NEC as well as a shorter antibiotic exposure as a result of lower LOS frequency among infants [114]. Furthermore, infants receiving probiotics, prebiotics, or symbiotics had lower clinical nosocomial sepsis rates and mortality rates than the placebo group [114]. However, two studies by Costeloe et al. [21,115] found no evidence of benefits for the routine use of *B. breve* BBG-001 for the prevention of NEC, LOS, and death in preterm infants (Table 1).

Notably, *B. infantis* is a crucial neonatal gut microbe that drives neonatal gut health. *B. infantis* allows infants to develop a healthy gut microbiome capable of metabolizing HMOs, while concurrently promoting bifidobacterial colonization, decreasing the abundance of taxa linked to preterm neonatal morbidities, and lowering antibiotic-resistant gene levels. To fully make use of the benefits of human milk, especially HMOs, a healthy gut microbiome is important. *B. infantis* forms a symbiotic relationship with its human host, providing protective benefits to both preterm and term infants while supporting the development of a healthy gut microbiota before weaning [98]. *B. infantis* is uniquely equipped at the genetic level to utilize the full range of HMO structures, making it the most complete and efficient HMO-utilizing organism capable of completely metabolizing these compounds [116]. Findings by Nguyen et al. [117] demonstrated that *B. infantis* EVC001 was needed to improve the functions required for obtaining extra energy from breast milk and limiting bacterial populations linked with poor growth and dysbiosis (example: *Enterobacteriaceae*, *Klebsiella*). Furthermore, there was also an increase in *Bifidobacterium* colonization in these preterm infants’ gut microbiomes. Moreover, they also found significantly lower antibiotic exposure in *B. infantis* EVC001-fed preterm infants. These *B. infantis* EVC001-fed infants had lower antibiotic resistance genes (ARGs), with a total drop in the resistome of 80.6% as compared to the control group. Additionally, multi-drug resistance genes were 227-fold higher on average in the control group. In this study, most of the ARGs recognized were present in potentially pathogenic species such as *Enterobacter* spp. and *Klebsiella* spp., which cause nosocomial infections and morbidities, including NEC and LOS. Despite finding unique ARG signatures transferred to infants, and that rapid acquisition of site-specific ARGs was observed [117], modulating the gut microbiome via *B. infantis* EVC001, has been demonstrated to be effective in combating the spread of ARGs in both term [118] and preterm infants, and was unlikely to result in novel resistance mechanism development [117]. Thus, *B. infantis* EVC001 administration may be beneficial by decreasing the spread of ARGs in pathogens causing NEC and LOS via modulating the intestinal microbiome. Furthermore, it was also found that supplementation of breastfed infants with *B. infantis* EVC001 significantly lowered fecal pH compared to controls, suggesting that this reduced pH played a crucial role in enhancing colonization resistance by inhibiting the growth and invasion of pathogenic bacteria in the infant gut [116].

Beyond these benefits, *B. infantis* also plays a crucial role in reducing enteric inflammation in the neonatal gut. As mentioned earlier, increased inflammatory responses have been implicated in NEC. The intestinal tract of preterm infants is known to be functionally immature. Studies have shown that the innate immune profile within the immature intestine is disproportionately skewed toward a pro-inflammatory state, with an imbalance between pro- and anti-inflammatory mediators [50,119]. Importantly, Nguyen et al. [117] found that *B. infantis* EVC001 seemed to be associated with a reduction in enteric inflammation. Although the role of *B. infantis* in decreasing enteric inflammation is not fully explained, earlier studies have demonstrated that *B. infantis* colonization enhances binding affinity and decreases inflammation in intestinal epithelial cells under in vivo conditions [120,121]. Furthermore, the normal development of immune tolerance has been linked with key *Bifidobacterium* species, particularly *B. infantis*, which may have the ability to normalize intestinal mucosa permeability [117,122]. This may in part explain the findings demonstrating a reduction in enteric cytokine production in preterm [117] and term infants [121] fed with *B. infantis* EVC001. Another possible explanation for the reduction in enteric inflammation upon administering *B. infantis* EVC001 may be a reduction in pathogenic bacteria abundance linked to higher endotoxin levels [123]. In addition, there are data suggesting that metabolites produced by *B. infantis* may directly modulate intestinal inflammation [121]. A study by Ehrlich et al. [124] found that *B. infantis* grown on HMOs produced high levels of the tryptophan metabolite indole-3-lactic acid (ILA), which was also elevated in the feces of infants with abundant *Bifidobacterium*. ILA significantly reduced LPS-induced NF-κB activation and IL-8 production in macrophages and intestinal epithelial cells [124]. In addition, the abundance of *B. infantis* EVC001, regardless of microbiome composition, corresponded to a drop in pro-inflammatory cytokine profiles [117], which may support the hypothesis that *B. infantis*-derived bacterial metabolites produced via HMO utilization induce the gut’s immune tolerance [124,125]. Hence, the use of *B. infantis* may have the potential to protect against excessive intestinal inflammation, possibly contributing to the prevention and/or treatment of NEC in preterm infants [98].

Importantly, the effects of probiotics are species- and strain-specific. Among *Bifidobacterium* species, the current evidence suggests that *B. infantis* may provide notable benefits in preterm infants, highlighting the importance of strain selection [126]. However, interpreting the findings across clinical trials remains challenging, as probiotic formulations are often poorly characterized, and their actual composition may differ from what is reported [127]. The wide variability in formulations and strains used in clinical studies highlights the challenges in comparing outcomes and the importance of accurate strain identification in both research and clinical practices [128].

#### 4.1.2. *Lactobacillus* spp.

Numerous studies have been conducted on *Lactobacillus* spp., particularly *Lactobacillus reuteri* (reclassified as *Limosilactobacillus reuteri* [129]) and *Lactobacillus rhamnosus* (reclassified as *Lacticaseibacillus rhamnosus* [129]) for the use of probiotics in preterm infants [130,131,132,133,134,135] (Table 1).

In terms of NEC, the findings on *L. reuteri* have shown limited efficacy to date. A study by Escárate et al. [130] found that *L. reuteri* administered in a single dose of 1 × 10^8^ CFU until 36 weeks of corrected gestational age did not affect NEC incidence, but it was associated with a decrease in severity, fatality rate, and surgical treatment requirement. Not all studies were as supportive, as Kaban et al. [131] found the prevalence of NEC (stages II and III) (0% vs. 6.4%) and mortality rates (2.1% vs. 8.5%) were lower in the *L. reuteri*-treated group—10^8^ CFU/day for ≥7 days or until infant discharged, experienced NEC, or died—than in the placebo group, but the mortality rate did not reach significance. Lastly, findings by Oncel et al. [132] demonstrated no statistically significant difference between infants fed *L. reuteri*, at a dose of 1 × 10^8^ CFU once a day until discharge or death, and the control group, which were given a placebo, with the frequency of NEC stage ≥ II (4% vs. 5%) and the overall rates of NEC and/or mortality (10% vs. 13.5%); however, there was a significantly lower frequency of proven sepsis in the probiotic group compared to the control group (6.5% vs. 12.5%) [132].

With regard to probiotics using the strain *L. rhamnosus*, Bonsante et al. [133] found *L. rhamnosus* LCR 35, as a prophylactic treatment administered twice daily at a dose of 2 × 10^8^ cells per unit until corrected GA of 36 weeks or at discharge, was associated with a significantly lower rate of NEC, mortality, and LOS. However, two studies found that *Lactobacillus* spp. probiotics did not provide benefits in terms of NEC and did not support their use in preterm infants. One was a randomized controlled study on preterm infants in Nepal by Dongol Singh et al. [134], which showed that although the probiotic *L. rhamnosus* LCR 35, administered at 0.4 mg (infants < 1500 g) and 0.8 mg (infants > 1500 g) in expressed breast milk twice daily until full feeding, did decrease NEC incidence by 12.35%, the difference was not significant. Hence, it could not demonstrate a trend in NEC reduction [134]. Another study by Kane et al. [135] conducted in the United States in fact showed that multivariable analysis demonstrated an increased risk of NEC after *L. rhamnosus* GG (ATCC 53103; LGG) supplementation at a dose of 2.5 × 10^9^ CFU/day and then increased to 5 × 10^9^ CFU/day until 35 weeks postmenstrual age, concluding that routine LGG supplementation was not associated with a decreased risk of NEC (Table 1).

#### 4.1.3. Other Probiotics: *Bacillus clausii* and *Saccharomyces boulardii*

Numerous studies have investigated the potential of *B. clausii* and *S. boulardii* as probiotics for the prevention and/or treatment of NEC and sepsis in preterm infants [136,137,138,139,140] (Table 1). The use of *B. clausii* as a probiotic has been conducted in India by Tewari et al. [136]; however, it was not able to provide a significant difference in LOS (blood culture-proven or probable) incidence, and also no difference in incidence of NEC was detected between the probiotic and placebo arm of both the extreme preterm infant and very preterm infant study groups.

The genus *Bacillus* is closely related to *Lactobacillus* as they both share the same class, *Bacilli*, under the phylum *Firmicutes*. *Bacillus* spp. consist of Gram-positive, spore-forming, rod-shaped aerobic or facultative anaerobic species, for instance, *B. clausii* [141]. This genus is generally soil inhabiting, but it can also be isolated from water, air, food, and even the gut of animals and humans [141]. Interestingly, under harsh environmental conditions, spore-forming bacteria can enter a dormant state by transforming into spores that are resistant to extreme temperature, pH conditions, harmful chemicals, and ultraviolet radiation and are able to survive nutrient and water deprivation. When conditions become favorable again, these spores germinate, giving rise to a new vegetative cell capable of growth, replication, and subsequent sporulation if needed [142,143]. Importantly, the spores of *Bacillus* are metabolically dormant and exhibit resilience to gastric acidity and bile salts. In addition, they demonstrate superior stability relative to vegetative cells during processing and storage of pharmaceutical or food-based probiotic formulations [141,142]. Potential mechanisms used by *B. clausii* to elicit their probiotic effects and promote gastrointestinal health include the immunomodulatory properties of immune cells; cytokine secretion and immunoglobulin levels; composition of gut microbiota; antimicrobial activity; effect on mucin production and mucosal barrier function; and resistance to multiple antibiotic classes [144].

Other than probiotics of bacterial origin, yeast-based probiotics have also been studied. *S. boulardii* is a type of yeast and represents a distinct species within the *Saccharomyces* genus [145]. It is the only yeast-based probiotic with scientific evidence [145,146]. Four studies explored *S. boulardii* as a probiotic in preterm infants, but the findings were inconsistent. Demirel et al. [140] found a significant reduction in the risk of clinical sepsis between the probiotic group administered a dose of 5 billion CFU of *S. boulardii* once a day, and the control group (34.8% vs. 47.8%). On the contrary, Serce et al. [137] and Xu et al. [138] found no reduction in sepsis incidence and no statistically significant difference in sepsis incidence. In terms of NEC, Demirel et al. [140] found no significant difference in death (3.7% vs. 3.6%) or NEC (4.4% vs. 5.1%) incidence in very low birth weight infants, while Serce et al. [137] found no significant difference in NEC or late-onset culture-proven sepsis incidence. Hence, both studies do not support the use of *S. boulardii* for NEC. Additionally, Park et al. [139] investigated the optimal time for initiating probiotics and found that the NEC frequency was higher in the late-initiated group (9.0%) than the early-initiated group (4.1%); however, the difference was not statistically significant. Nonetheless, they concluded that early initiation of *S. boulardii* within a week after birth may decrease the risk of adverse outcomes among preterm infants [139] (Table 1). Hence, it is worth noting that the timing of administering probiotics may contribute to NEC outcomes.

*S. boulardii* demonstrates several characteristics that support its potential as a probiotic. For instance, it is capable of withstanding gastrointestinal transit, exhibits an optimal growth temperature of 37 °C under both in vitro and in vivo conditions, and has the ability to inhibit the growth of various microbial pathogens [146]. The mechanisms of action used by *S. boulardii* include antitoxin, antimicrobial, cross-talk with normal microbiota, trophic action on the intestinal mucosa, and regulation of immune response (acting as an immune stimulant, decreasing pro-inflammatory response, and increasing mucosal anti-inflammatory signaling effects) [147]. *S. boulardii* has been used in numerous countries as both a preventive and therapeutic agent for diarrhea and other gastrointestinal disorders associated with the use of antimicrobial agents [146]. *S. boulardii* is effective as an adjunct therapy in the treatment of both acute and chronic gastrointestinal diseases; for instance, diarrhea and irritable bowel syndrome (IBS) [145,146,147]. These findings highlight the need for further research on its use in preterm infants.

**Table 1:** The application of single-strain probiotics in preterm infants, focusing on its effect on necrotizing enterocolitis (NEC), sepsis, and mortality.

Probiotic	Dosage/Type of Feeding	Probiotic Treatment Period	Sample Size, Gestational Age (GA^b^), Birth Weight (BW^c^)	Country	NEC/Sepsis Findings	Study
*Bifidobacterium infantis* EVC001 (Evivo^TM^, Evolve BioSystems^+^, Davis*, California*, USA*)	A total of 8 billion CFU^a^. Suspended in 0.5 mL of medium-chain triglyceride oil daily via gastric tube before a morning feed. Feeding volumes of 80–100 mL/kg/day. Mother’s milk/donor milk/both. Bovine milk-based formula/bovine milk-based human milk fortifier/human milk-based fortification/bovine milk-based fortification.	From June 2018 to July 2019. In August 2019, the administration protocol was revised to begin on the second day of trophic feeding. EVC001 administration continued until 34 weeks postmenstrual age or for a minimum of 2 weeks, whichever was longer.	Sample size: *n* = 483 (probiotics: *n* = 182; no probiotics: *n* = 301) GA (mean): 28 weeks BW < 1500 g	USA	NEC and mortality: Cumulative incidence of NEC in infants fed with more than one dose of *B. infantis* EVC001 dropped from 11% to 2.7% (*p* < 0.01), a 73% risk reduction (*p* < 0.01). NEC-associated mortality dropped from 2.7% to 0% (*p* = 0.03) when comparing unexposed cohort to exposed cohort. No adverse effects.	[112]
*Bifidobacterium breve* M-16V (Morinaga Milk Industry Co., Ltd., Tokyo*, Japan)	A total of 3 × 10^9^ (3 billion) CFU/day. For neonates < 28 weeks, the daily dose was 1.5 × 10^9^ CFU/day until reaching feeds of 50 mL/kg/day, and then increased to 3 × 10^9^ CFU/day. Breast milk (first choice) or sterile water for injection was used for reconstitution of the dry powder in 1 g sachets.	The probiotic supplementation was started when infants were ready for enteral feeds and continued until the corrected age of 37 weeks. Probiotics stopped when feeds were stopped by the attending neonatologist for indications such as sepsis and NEC.	Sample size: *n* = 1755 (before probiotics: *n* = 835; after probiotics: *n* = 920) GA < 34 weeks BW (median): 1340 g	Australia	NEC and/or mortality: Significant decrease in NEC ≥ stage II and the composite outcome of ‘NEC ≥ stage II or all-cause mortality’ in neonates < 34 weeks.	[113]
*Bifidobacterium lactis* (Maflor, Mamsel Pharmaceutical Company, Istanbul, Turkey)	Three study groups: *Bifidobacterium lactis* (5 × 10^9^ CFU). Prebiotic (inulin, 900 mg). Synbiotic (*Bifidobacterium lactis*, 5 × 10^9^ CFU, 30 mg plus inulin, 900 mg). One sachet per day with breast milk or formula milk.	Intervention began in parallel with enteral feeding (within <24 h) until infant was discharged or died, whichever came first, for a maximum of 8 weeks.	Sample size: *n* = 400 (probiotics: *n* = 100; prebiotic: *n* = 100; synbiotic: *n* = 100; placebo: *n* = 100) GA < 32 weeks BW < 1500 g	Turkey	NEC: Lower NEC rates in probiotic (2%) and symbiotic (4%) groups than placebo (18%) and prebiotic groups (12%) (*p* < 0.001). *Bifidobacterium lactis*, alone or in combination with prebiotics (inulin), improved infants’ resistance to NEC. Sepsis and Mortality: *Bifidobacterium lactis* and prebiotics, alone or in combination, demonstrated lower late-onset sepsis (LOS) frequency, and thus, a shorter antibiotic exposure. Lower rates of clinical nosocomial sepsis (*p* = 0.004) and mortality rates (*p* = 0.003) for infants receiving probiotics, prebiotics, or symbiotics than the placebo.	[114]
*Bifidobacterium breve* BBG-001	A total of 1 mL of *Bifidobacterium breve* BBG-001 in 1/8 strength infant formula (6.7 × 10^7^ to 6.7 × 10^9^ CFU) per dose. Breast milk/formula milk, maternal breast milk encouraged, but feeding regimes were not standardized.	Started as soon as practicable and continued daily until 36 weeks postmenstrual age.	Sample size: *n* = 1310 (probiotics: *n* = 650; placebo: *n* = 660) GA: 23 –30 weeks BW (median): 1010 g (49% < 1000 g)	United Kingdom	NEC, LOS, and mortality: No benefit for NEC (10% vs. 9.4%), LOS (11.7% vs. 11.2%), and death (8.5% vs. 8.3%) in preterm infants. Did not support routine use of probiotics for preterm infants.	[21,115]
*Lactobacillus* *reuteri Protectis* (LRP), DSM 17938 strain (BioGaia^®^ AB, Stockholm*, Sweden*)	In a single dose (1 × 10^8^ CFU). Breast milk is preferably used, as well as preterm formula or a combination of both; fortified breast milk.	Probiotics started between the third and seventh day of life until 36 weeks of corrected GA.	Sample size: *n* = 772 (probiotics: *n* = 319; controls (no probiotics): *n* = 453) GA < 32 weeks BW < 1500 g	Chile	NEC: No effect on NEC incidence.	[130]
*Lactobacillus* *reuteri* DSM 17938 suspension (Interlac^®^) (BioGaia^®^ AB, Stockholm, Sweden)*	A total of 10^8^ CFU/day. Breast milk and/or formula.	Probiotics or placeboes given as soon as the neonate reached a stable condition, i.e., could be orally or enterally fed breast milk and/or formula at least 40–50 mL/kg body weight and had two sequential feedings of 40–50 mL/kg body weight. Intervention administered for ≥7 days, or until infant discharged, experienced NEC, or died.	Sample size: *n* = 94 (probiotics: *n* = 47; placebo: *n* = 47) GA: 28–34 weeks BW: 1000–1800 g	Indonesia	NEC and mortality: Lower prevalence of NEC stages II and III (0% vs. 6.4%) and mortality rates (2.1% vs. 8.5%) in the probiotic group than the control group, but the findings were insignificant. Insignificant mortality rate between groups. Safe.	[131]
*Lactobacillus* *reuteri* DSM 17938 (Biogaia^®^ AB, Stockholm, Sweden)	Fed with oil-based suspension containing 1 × 10^8^ CFU. Once a day mixed in breast milk or formula.	Intervention started from first feeding until discharge or death.	Sample size: *n* = 400 (probiotics: *n* = 200; placebo: *n* = 200) GA ≤ 32 weeks BW ≤ 1500 g	Turkey	NEC, sepsis, and mortality: No statistically significant difference between probiotic and placebo groups in terms of frequency of NEC stage ≥ II (4% vs. 5%) or overall NEC or mortality rates (10% vs. 13.5%). Significantly lower frequency of proven sepsis in the probiotic group compared to the placebo group (6.5% vs. 12.5%).	[132]
*Lactobacillus casei rhamnosus* LCR 35 (*Lcr Restituo*) (Probionov, France)	Two daily administrations of 2 × 10^8^ cells per unit of *Lactobacillus casei rhamnosus* LCR 35 (*Lcr Restituo*). Exclusively breast milk then human milk fortification.	Probiotics started as soon as minimal enteral feeding commenced until corrected GA of 36 weeks or at discharge.	Sample size: *n* = 1130 (probiotics: *n* = 347; historical: *n* = 783) GA: 24 –31 weeks BW: 1220 ± 353 g (probiotic); 1214 ± 329 g (historical)	France	NEC, LOS, and mortality: Significantly lower rates of NEC, mortality, and LOS. Occurrence of LOS was significantly delayed. No significant adverse effects.	[133]
*Lactobacillus casei rhamnosus* (LCR 35)	At a dose of 0.8 mg (infants > 1500 g) and 0.4 mg (infants < 1500 g) in 2 mL and 1 mL of expressed breast milk, respectively, twice daily.	Probiotics started on the second day of life until they reached full feeding.	Sample size: *n* = 72 (probiotics: *n* = 37; placebo: *n* = 35) GA (mean): 32.6 ± 2.2 weeks BW < 2000 g	Nepal	NEC: NEC incidence reduced by 12.35% between probiotic and placebo groups, but was not significant. Unable to demonstrate a trend in the direction of NEC reduction.	[134]
*Lactobacillus rhamnosus* GG ATCC 53103 (LGG) (Culturelle^®^, i-Health, Cromwell, CT, USA)	Once daily at a dose of 2.5 × 10^9^ CFU/day, then increased to 5 × 10^9^ CFU/day. For infants feeding 1–2 mL every 3 h (or an equivalent hourly volume), LGG^f^ was mixed in sterile water. For infants feeding 3 mL every 3 h or greater (or an equivalent hourly volume), LGG was mixed in either breast milk or formula.	Probiotics initiated once an infant was tolerating enteral feeding until 35 weeks postmenstrual age.	Sample size: *n* = 644 (probiotics: *n* = 175; no probiotics: *n* = 465) GA (median): 28.7 weeks BW < 1500 g	USA	NEC: Multivariable analysis showed an increased NEC risk after LGG supplementation. Routine LGG supplementation was not associated with a decreased risk of NEC.	[135]
*Bacillus clausii* (Enterogermina^®^, Sanofi-Aventis, Italy)	5 mL mini-bottles containing 2 × 10^9^ spores. In a dose of 2 mL per-oral every 8 h, mixed with the enteral feeds through orogastric tube or oral feeds to give them 2.4 × 10^9^ spores per day. Expressed/donor breast milk.	Intervention was started typically by day 5 in asymptomatic and day 10 in symptomatic neonates. Probiotics administered until infant reached postnatal age of 6 weeks, or till discharge, death, or occurrence of LOS, whichever was earlier.	Sample size: Extreme preterm: *n* = 120 (probiotics: *n* = 61; placebo: *n* = 59) Very preterm: *n* = 124 (probiotics: *n* = 62; placebo: *n* = 62) GA < 34 weeks BW < 2500 g	India	NEC: No difference in NEC incidence between probiotic and placebo arms in both groups. LOS: No significant difference in blood culture-proven or probable LOS incidence between the probiotic and placebo arms for the extreme preterm infant group (23% vs. 29%) and the very preterm infant group (10% vs. 13%).	[136]
*Saccharomyces boulardii* (Reflorâ, Biocodex, Beauvais, France)	Once a day, 250 mg (5 billion CFU) was added to breast milk or formula.	Starting with the first feed until discharged.	Sample size: *n* = 271 (probiotics: *n* = 135; no probiotics: *n* = 136) GA ≤ 32 weeks BW ≤ 1500 g	Turkey	NEC and mortality: Ineffective at reducing incidence of death (3.7% vs. 3.6%) between the probiotic and control groups. Ineffective at reducing incidence of NEC (4.4% vs. 5.1%) between the probiotic and control groups. Clinical sepsis: Reduced incidence of clinical sepsis in the probiotic group; significant difference in rate of clinical sepsis between probiotic and control groups (34.8% vs. 47.8%); and reduced culture-negative sepsis.	[140]
*Saccharomyces boulardii* (Reflor^®^, Biocodex, France)	A total of 50 mg/kg equal to 0.5 × 10^9^ cell/kg per dose twice daily. Breast milk or formula.	Starting with the first feed until discharged.	Sample size: *n* = 208 (probiotics: *n* = 104; placebo: *n* = 104) GA ≤ 32 weeks BW ≤ 1500 g	Turkey	NEC, LOS, and mortality: No decrease in incidence of NEC or sepsis. No significance difference in the incidence of stage ≥ II NEC or death. No significant difference in the incidence of stage ≥ II NEC or late-onset culture-proven sepsis.	[137]
*Saccharomyces boulardii* CNCM I-745 (Bioflor^®^, CMS Shenzhen Kangzhe Pharmaceutical Co. Ltd., Shenzhen, China; manufactured by Biocodex, Paris, France)	Twice a day as separate medication, not mixed with formula at a dosage of 50 mg/kg (50 mg was approximately 10^9^ CFU). No infant received mother’s milk.	Minimal duration of the intervention was ≥7 days. Probiotics were given until the 28th day after birth or when the infant was discharged from the hospital.	Sample size: *n* = 125 (probiotics: *n* = 63; no probiotics: *n* = 62) GA: 30–37 weeks BW: 1500–2500 g	China	Sepsis: No statistically significant difference in sepsis incidence. No adverse effects.	[138]
*Saccharomyces boulardii* CNCM I-745 (Bioflor 250 powder^®^, Kuhnil, Seoul*, South Korea*)	A probiotic of 5 × 10^9^ CFU was administered with breast milk or formula milk twice a day.	Supplementation starting time was determined by the pediatricians or neonatologists.	Sample size: *n* = 370 (early introduction (within 7 days of birth): 147; late introduction (after 7 days of birth): 223) GA < 32 weeks BW < 1500 g	South Korea	NEC: NEC frequency was higher in the late-initiated group (9.1%) than the early-initiated group (4.1%), but the difference was not statistically significant. Early initiation of *Saccharomyces boulardii* within a week after birth may decrease the risk of adverse outcomes among preterm infants.	[139]

^+^ Evolve Biosystems has been changed to Infinant Health; * The information on countries for probiotics products was obtained through respective official websites; ^a^ Colony-forming unit; ^b^ Gestational age; ^c^ Birth weight.

### 4.2. Multiple-Strain Probiotics

#### 4.2.1. Dual-Strain Probiotics

Many studies investigated combination probiotics by mixing two of the most common probiotic genera, *Lactobacillus* spp. and *Bifidobacterium* spp., to evaluate their potential in the prevention and/or treatment of NEC and/or LOS in preterm infants (Table 2).

In Bangladesh, Chowdhury et al. [148] reported that VLBW infants administered a combination of *Bifidobacterium* spp. and *Lactobacillus* along with breast milk had significantly lower developments of NEC than the negative control group (1.9% vs. 11.5%), concluding that probiotic supplementation reduced NEC frequency in VLBW preterm infants [148]. However, the findings of Chowdhury et al. [148] were inconsistent to those of Lambaek et al. [149], and Dang et al. [150]. The study from Denmark by Lambaek et al. [149], demonstrated that *B. lactis* (1 × 10^8^) and LGG (1 × 10^9^) probiotics combination had no significant reduction in the risks of NEC or mortality. In terms of NEC outcomes, this finding was consistent with Dang et al. [150], who studied the use of probiotics combination containing a daily dose of 500 million CFU/species of LGG and *B. infantis* on preterm infants. Although Dang et al. [150] showed the probiotics group had significantly lower odds of extra-uterine growth restrictions, with the odds being 70% lower in the adjusted model, no significant difference was found in NEC incidence between study groups. Regarding sepsis, Fortmann et al. [151] studied the effects of combination probiotics *Lactobacillus acidophilus* and *B. infantis* (daily dose containing 1–3 × 10^9^ CFU and 1–1.5 × 10^9^ CFU respectively) in preterm infants on a range of feeds, finding that probiotics administered to infants fed exclusively human milk had the lowest clinical sepsis incidence (34%) compared to infants in the group on a combination of human milk and formula milk (35.5%) and formula group (40%). This study found that probiotic supplementation proved to be protective against clinical sepsis only in the mixed feeds group [151].

Additionally, a study reported on the effect of a combination probiotic on bacterial colonization. In a multicenter randomized double-blind placebo-controlled trial conducted in Poland, Strus et al. [152] used a combination of *L. rhamnosus* KL53A and *B. breve* PB04 in preterm infants. The combination probiotic effectively colonized the gut, rebalancing the distorted gut microbiota, and it may play a part in decreasing the Staphylococcus sepsis rate. Stool analysis showed probiotic administration was linked with significantly higher populations of *L. rhamnosus* and *B. breve* compared to other cultivable bacteria, particularly the potentially pathogenic bacteria, predominantly in the first weeks of life. Molecular analysis of stool samples indicated that *L. rhamnosus* KL53A and *B. breve* PB04 colonization in the study group were 98% and 94%, respectively. In fact, throughout the study period, 91% of infants were colonized by both strains [152]. Overall analysis of cohort showed that the incidence of staphylococcal sepsis was lower in the placebo group with *B. breve* colonization [152].

One of the common dual-stain probiotics that has been studied is Infloran, a combination consisting of *L. acidophilus* and *Bifidobacterium* spp. (either *B. bifidum* or *B. infantis*). Outcomes on NEC using Infloran have been inconsistent. Healy et al. [153] administered Infloran (*L. acidophilus* and *B. bifidum*) capsules containing no less than 10^9^ CFU to preterm infants at a daily dose of 250 mg/kg until 34 weeks corrected gestational age, and they found significant reductions in the incidence of the combined outcomes of death or severe NEC after routine administration of the probiotic supplement. However, a study conducted in Thailand by Saengtawesin et al. [154], which administered the same probiotic combination with the same dosage to preterm infants as Healy et al. [153], found no effect on NEC incidence. Additionally, in a single retrospective study by Cripps et al. [155], very preterm infants were fed with either Infloran (*n* = 361) (*B. bifidum* and *L. acidophilus*) or ABC Dophilus (*n* = 25) (*B. infantis*, *Streptococcus thermophilus*, and *B. lactis* with 1 × 10^9^ total organisms in 1.5 g) probiotics had a lower incidence of NEC, LOS, and mortality than the group without probiotics after adjusting for confounders in multivariate analysis. Additionally, Denkel et al. [156] administered Infloran (*L. acidophilus* and *B. infantis*) to preterm infants as routine prophylaxis and found significant reductions in NEC, overall mortality, and mortality after NEC. In terms of NEC, this was consistent with findings by Repa et al. [157], who used the same probiotic combination and found a significant reduction in NEC in the subgroup analysis; however, this was observed only in infants fed with breast milk, while this probiotic was not effective in infants fed formula milk exclusively. Hence, this suggested an association between the type of feeding and probiotics efficacy, which is in agreement with Samuels et al. [158], who found Infloran (*L. acidophilus* and *B. bifidum*) showed decreased adjusted odds for the composite outcome (sepsis, NEC, mortality) only in infants fed breast milk exclusively. Nonetheless, Samuels et al. [158] concluded that the introduction of probiotics was not associated with a reduction in ‘NEC or death’ and that the type of feeding seemed to modify the effects of probiotics (Table 2). In these studies, the reduction in NEC and/or sepsis was observed only in the probiotics-fed group that was fed breast milk, as breast milk contains *Bifidobacterium* and *Lactobacillus* that readily colonize the gastrointestinal tract of preterm infants [159,160,161]. Furthermore, milk oligosaccharides found in breast milk are thought to promote the growth of beneficial gut microbiota—*Bifidobacterium* [162].

It is, however, of note that breastfeeding seemed to have weak associations with the levels of gut *Lactobacillus* [163]. This suggested its effect may instead be indirect, involving synergistic interaction that aids the growth of *Lactobacillus* and *Bifidobacterium* in the preterm infant gut. For instance, studies have suggested that the prebiotic substrate galacto-oligosaccharides (GOS) exhibited a growth-promoting ability for certain *Lactobacillus* and *Bifidobacterium* strains [164,165]. GOS are non-digestible oligosaccharides comprising a mix of structures that differ in their degree of polymerization and the type of glycosidic bonds between linking galactose moieties and connecting galactose to glucose [166]. As one of the most extensively studied prebiotics, their beneficial effects are well-established [165]. GOS can be synthesized from soyabeans and lactose (from cow’s milk), with the latter resembling oligosaccharides in human breast milk [167]. This provides a theoretical basis for the reason why breastfeeding, together with probiotic administration, promotes beneficial bacteria *Bifidobacterium* and *Lactobacillus* growth, reducing the risk of NEC.

Furthermore, an additional study reported that Infloran significantly affected the gut microbiome composition. Chang et al. [168] found significantly different microbiota profiles in preterm infants fed with probiotics. Probiotic supplementation was shown to significantly boost the abundance of probiotic genera while decreasing the pathogenic bacteria in preterm infants from 2 weeks of age until discharge. In addition, the beta diversity analysis showed differences in the microbial composition between the two groups after 1 month of age. Compared to the control group, preterm infants fed Infloran showed a significantly higher abundance of probiotic genera of *Bifidobacterium* and *Lactobacillus* on days 14, 30, and 60, and decreased pathogenic bacteria of *Klebsiella* on days 14 and 30 and *Escherichia*–*Shigella* on day 60 [168]. The control group had a higher proportion of preterm infants with a low abundance (<1%) of *Bifidobacterium* or *Lactobacillus*. Beta diversity was also significantly different between the probiotic group and the control group on days 30 and 60 [168]. In preterm infants, delayed *Bifidobacterium* colonization, along with a reduced abundance of *Bifidobacterium*, may contribute to NEC pathogenesis [168,169,170,171,172,173]. Hence, Infloran may modulate the gut microbiota of preterm infants, leading to a healthy microbial community [168] and thus potentially reducing the risks of NEC and LOS in preterm infants.

**Table 2:** The use of dual-strain probiotics in preterm infants, focusing on its effect on necrotizing enterocolitis (NEC), sepsis, and mortality.

Probiotic	Dosage/Type of Feeding	Probiotic Treatment Period	Sample Size, Gestational Age (GA^b^), Birth Weight (BW^c^)	Country	NEC/Sepsis Findings	Study
*Bifidobacterium* spp., *Lactobacillus* (manufactured by Tensall Bio-tech Co. Ltd., DungShan Township*, Yilan 269*, Taiwan; distributed by Century Health Care, Bangladesh)	Per capsule contains *Bifidobacterium* spp., *Lactobacillus* at 6 × 10^9^ CFU^a^ = 6 billion CFU. A total of 3 × 10^9^ CFU/day. Once daily with breast milk from first feeding through nasogastric tube until discharged (continued for at least 10 days). Probiotics were given after 2 h of giving intravenous antibiotic.	From first feeding (after trophic feeding) through nasogastric tube until discharged (continued for at least 10 days).	Sample size: *n* = 102 (probiotics: *n* = 52; no probiotics: *n* = 50) GA: 28–33 weeks BW: 1000–1499 g	Bangladesh	NEC: Development of NEC was significantly lower in the study group than the control group (1.9% vs. 11.9%). Reduced NEC frequency in very low birth weight infants.	[148]
*Lactobacillus acidophilus*, *Bifidobacterium infantis*	Daily dose contained 1–3 × 10^9^ CFU *Lactobacillus acidophilus* and 1–1.5 × 10^9^ *Bifidobacterium infantis*. Provided once or twice daily in capsules. Human milk/mixed (combination of human milk and formula milk)/formula.	Starting from day 1 to 3 of life until day 28 of life.	Sample size: *n* = 7516 (exclusively human milk: *n* = 1568; mix: *n* = 5221; exclusively formula: *n* = 727) GA: 22 + 0–28 + 6 weeks and/or BW < 1500 g	Germany	Sepsis: Human milk group had the lowest incidence of clinical sepsis (34.0%) compared to the mix group (35.5%) and the formula group (40.0%). Only in the mix group, probiotic supplementation proved to be protective against clinical sepsis.	[151]
*Bifidobacterium lactis* BB12, *Lactobacillus rhamnosus* GG	Bifiform capsules containing *Bifidobacterium lactis* BB12 1 × 10^8^ and *Lactobacillus rhamnosus* GG 1 × 10^9^. Contents of two capsules dissolved in milk. Once daily via nasogastric tube to infants completed less than 30 weeks of gestation, starting from the third day of life. If the infant had 1 mL of milk per meal or more, probiotics were added, otherwise not. Unpasteurized maternal/donor milk.	Probiotics were given starting from the third day of life until discharge from hospital.	Sample size: *n* = 714 (probiotics: *n* = 333; before probiotics: *n* = 381) GA < 30 weeks (mean GA 27.1 ± 1.7 weeks) BW: Not available (mean birth weight was 935 ± 381 g)	Denmark	NEC and mortality: No statistically significant reduction in the risks of NEC or mortality.	[149]
*Lactobacillus rhamnosus* GG (LGG) (Culturelle, Amerifit Brand, Cromwell, CT, USA), *Bifidobacterium infantis* (Align, Procter and Gamble, Cincinnati, OH, USA)	Totals of 500 million CFU of *Lactobacillus rhamnosus* GG (LGG) and 500 million CFU of *Bifidobacterium infantis*. One capsule of Culterelle (10 billion CFU) and 10 capsules of Align (10 billion CFU) were mixed in 20 mL of Similac Special Care (Abbott Nutrition, Columbus, OH, USA) 20 kilocalories per oz formula. A total of 1 billion CFU (500 million CFU of each species) in every milliliter. One milliliter of prepared probiotics supplementation was added to the first enteral feeding and continued once daily with feedings. Breast milk/fortification of breast milk/formula.	Started from first enteral feeding and continued until 34 weeks postmenstrual age.	Sample size: *n* = 221 (after probiotics: *n* = 108; before probiotics: *n* = 113) GA ≤ 28 weeks and/or BW ≤ 1250 g	United Kingdom	NEC: No significant difference in the incidence of NEC in the adjusted model.	[150]
*Bifidobacterium bifidum* and *Lactobacillus acidophilus* (Infloran^®^, [Desma Healthcare] (Laboratorio Farmaceutico S.I.T.S.r.l., Pavia, Italy)*	Daily dose of 250 mg/kg. Each 250 mg capsule of probiotic contained no less than 10^9^ CFUs each of *Bifidobacterium bifidum* and *Lactobacillus acidophilus*. Sufficient volume of milk was being administered to dissolve the probiotic granules.	Started once infant was deemed to tolerate enteral feeds and sufficient volume of milk was administered (to dissolve probiotic granules). Discontinued at 34 weeks corrected GA.	Sample size: *n* = 744 (probiotics: 353; pre-probiotics: 391) GA < 32 weeks BW < 1500 g	Ireland	NEC or mortality: Significant reductions in the incidence of combined outcomes of death or severe NEC after routine administration of probiotic supplement.	[153]
*Lactobacillus acidophilus* and *Bifidobacterium bifidum* (Infloran^®^) (Laboratorio Farmaceutico S.I.T.S.r.l., Pavia, Italy)*	*Lactobacillus acidophilus* 1 × 10^9^ and *Bifidobacterium bifidum* 1 × 10^9^ organisms. 125 mg/kg/dose twice a day with breast milk or premature formula.	From the start of feeding until 6 weeks or discharge.	Sample size: *n* = 60 (probiotics: *n* = 31; no probiotics: *n* = 29) GA ≤ 34 weeks BW ≤ 1500 g	Thailand	NEC: No effect on NEC incidence.	[154]
ABC Dophilus (Solgar, Leonia, NJ, USA): *Bifidobacterium infantis*, *Streptococcus thermophilus* and *Bifidobacterium lactis* Infloran^®^ (Laboratorio Farmaceutico S.I.T.S.r.l., Mede, Italy): *Bifidobacterium bifidum* and *Lactobacillus acidophilus*	ABC Dophilus: *Bifidobacterium infantis*, *Streptococcus thermophilus* and *Bifidobacterium lactis* with 1 × 10^9^ total organisms in 1.5 g. Doses of 750 mg of probiotic powder twice daily. Infloran: 2 × 10^9^ of *Bifidobacterium bifidum* and *Lactobacillus acidophilus* per 250 mg capsule. The dose was weight-dependent as infants < 750 g received 1/4 capsule twice daily; infants 750–1500 g received ½ capsule twice daily; and infants > 1500 g received one capsule twice daily. Breast milk/formula.	ABC Dophilus: Started once an infant received 3 mL of milk per feed until discharge or term corrected age. Infloran: Started once infant tolerated 1 mL of milk every 4 h for 24 h. Infloran ceased once infants were >2 kg or >34 weeks of corrected age.	Sample size: *n* = 805 (probiotics: ABC Dophilus: *n* = 25; Infloran: *n* = 361; no probiotics: *n* = 419) GA < 32 weeks BW < 1500 g	Australia	NEC, late-onset sepsis (LOS) and mortality: Univariate analysis: The probiotic group had a lower incidence of NEC (7.6% vs. 3.6%), and a reduction in the rate of LOS (22.4% vs. 14.2%) and mortality (9.5% vs. 4.6%) than the group without probiotics. Multivariate analysis: probiotic therapy in very low birth weight infants was associated with a lower incidence of NEC, LOS, and mortality.	[155]
*Lactobacillus acidophilus* and *Bifidobacterium infantis* (Infloran^®^) (Laboratorio Farmaceutico S.I.T.S.r.l., Mede, Italy)	Not available	Not available	Sample size: *n* = 10,890 (probiotics: *n* = 5818; no probiotics: *n* = 5072) GA: <27 weeks to >30 weeks BW < 1500 g	Germany	NEC and mortality: Infloran as a routine prophylaxis showed significant reductions in NEC incidences, overall mortality, and mortality after NEC.	[156]
*Lactobacillus acidophilus* and *Bifidobacterium infantis* (Infloran^®^) (Laboratorio Farmaceutico S.I.T.S.r.l., Sanremo, Italy)	One capsule contained 10^9^ *Lactobacillus acidophilus* and 10^9^ *Bifidobacterium infantis* Twice a day with enteral feedings. Breast milk/infant formula.	From birth until discharge or 37 weeks GA. Infloran was not used in infants with gut malformations and discontinued if definite NEC occurred.	Sample size: *n* = 463 (probiotics: *n* = 230; controls (no probiotics): *n* = 233) GA < 34 weeks BW < 1500 g	Europe	NEC: Multivariate analysis on the effect of probiotics according to the type of feeding: Significant reduction in NEC only in the probiotics group fed with breast milk. Ineffective in infants exclusively fed formula.	[157]
*Lactobacillus acidophilus* (ATCC 4356) and *Bifidobacterium bifidum* (ATCC 15696) (Infloran^®^) (Laboratorio Farmaceutico S.I.T.S.r.l., Mede, Italy)	250 mg capsules containing 10^9^ CFU *Lactobacillus acidophilus* (ATCC 4356) and 10^9^ CFU *Bifidobacterium bifidum* (ATCC 15696). Daily dose of one capsule dissolved in 2 mL of (breast or formula) milk and given per nasogastric tube.	Started at the first enteral feed of at least 1 mL per bolus and continued until 35 weeks postmenstrual age or until neonatal intensive care unit discharge, whichever came first.	Sample size: *n* = 1961 (after probiotics: *n* = 673; before probiotics: 1288) GA < 32 weeks BW < 1500 g	The Netherlands	NEC, sepsis, and mortality: Decreased adjusted odds for the composite outcome (sepsis, NEC, mortality) only in infants fed exclusively breast milk. Probiotics were not associated with a reduction in ‘NEC or death’, and that the type of feeding seemed to modify the effects of probiotics.	[158]

* The information on countries for probiotics products was obtained through respective official websites; ^a^ Colony-forming unit; ^b^ Gestational age; ^c^ Birth weight.

In summary, most studies showed that dual-strain probiotics have beneficial effects on NEC and/or LOS in preterm infants. Nevertheless, a few studies showed positive outcomes only in infants fed human breast milk, suggesting that breast milk may contribute to these effects.

#### 4.2.2. More than Two Strains of Probiotics

Several studies investigated the use of >2 probiotic strains for the prevention and/or treatment of NEC and/or LOS in preterm infants, typically combining *Bifidobacterium* spp., *Lactobacillus* spp., *Streptococcus* sp., or *Enterococcus* spp. (Table 3). *S. thermophilus* is the commonly used probiotic species of the genus *Streptococcus* in the following studies. *S. thermophilus* is part of the lactic acid bacteria clade. It is a Gram-positive bacterium classified within the phylum *Firmicutes*, genus *Streptococcus*, family *Streptococcaceae*, and order *Lactobacillales* [174,175]. Traditionally, *S. thermophilus* is used to make yogurt, and it is the only *Streptococcus* species used in the food industry that is also recognized as GRAS in the United States by the FDA [176]. It has also obtained the QPS status by the European Union [175,177]. The health benefits associated with *S. thermophilus* on host health include stimulation of the gut immune system, anti-inflammatory effects, the production of antioxidant compounds, antimutagenic effects, risk alleviation for some types of cancer, and antimicrobial activity [176,177]. Despite these benefits and its ability to produce at least 10^8^ CFU live starter microorganisms per gram of fermented product, its probiotic status is still under debate [176,177].

In terms of studies using probiotics combinations of three different strains, a study conducted in Bangladesh by Mannan et al. [178] found preterm infants fed with probiotic combination *L. acidophilus*, *Lactobacillus bulgaricus*, and *B. bifidum* added with fructo-oligosaccharides had significantly lower NEC incidence (1.7% vs. 13.3%) and mortality compared to the placebo group. In addition, both Chiruvolu et al. [179] and Jacobs et al. [180] studied the probiotic combination *B. infantis*, *Streptococcus thermophilus*, and *B. lactis*, but their findings were not fully in agreement. Chiruvolu et al. [179] found that although this probiotic combination showed decreased incidence of NEC from 6.3% in the group without probiotic to 1.6% in the probiotic group, the differences in NEC, LOS, and death were not significant after adjusting for multiple variables. On the contrary, Jacobs et al. [180] demonstrated that this combination resulted in a significant reduction in NEC of Bell’s stage ≥ II, but no difference in definite LOS or all-cause mortality. The findings on LOS, however, were inconsistent with the findings of Kanic et al. [181], which used the combination probiotic *L. acidophilus* (subsp. *L. gasseri*), *B. infantis*, and *Enterococcus faecium* and found that it was beneficial in preventing late-onset infections and reduced LOS frequency. In terms of NEC, although Li et al. [182] studied the probiotic combination of *B. infantis*, *S. thermophilus*, and *B. bifidum*, and suggested it was safe to be used in preterm infants for NEC prevention, due to the observational study design, they were not able to detect any change in outcome. Additionally, Guney-Varal et al. [183] found that the probiotic group fed with a combination of four different probiotic strains (*L. rhamnosus* (4.1 × 10^8^ CFU) + *Lactobacillus casei* (reclassified as *Lacticaseibacillus casei* [129]) (8.2 × 10^8^ CFU) + *Lactobacillus plantarum* (reclassified as *Lactiplantibacillus plantarum* [129]) (4.1 × 10^8^ CFU) + *Bifidobacterium animalis* (4.1 × 10^8^ CFU)), at a high dosage and at a targeted period of 4 to 6 weeks along with prebiotics, had significantly lower NEC incidence and rate of mortality. Overall, the authors concluded that high doses and the prolonged use of combined multi-strain and multi-species probiotics showed beneficial effects on gastrointestinal complications, mortality, and sepsis-related mortality from NEC in preterm infants.

Interestingly, studies using four or more strains more consistently report benefits in preterm infants. For instance, a study by Roy et al. [184], conducted in India, showed probiotics with the combination *L. acidophilus* (1.25 billion), *B. longum* (0.125 billion), *B. bifidum* (0.125 billion), and *B. lactis* (1.0 billion) per 1 g sachet had the potential of lowering the rate of invasive fungal sepsis. They found that the occurrence of LOS (inclusive of fungal sepsis) was 55.4% in the probiotics group and 75% in the placebo group (*p* = 0.02). The authors believed that by reducing gut colonization due to *Candida* species would help to reduce the development of invasive fungal species. Their findings on colonization of *Candida* species, however, contradict the findings by Gray et al. [185], who investigated the use of four different probiotics consisting of one to three bacterial strains, and found increased odds of *Candida* infection in infants fed with probiotics. It is worth noting that *Candida* spp. are the third leading cause of neonatal LOS in low birth weight infants below 1500 g, with *C. parapsilosis* increasingly recognized as a major pathogen in neonates with central venous access [58]. In addition, *Candida* spp. infections are responsible for 6–18% of LOS cases in preterm infants admitted to the NICU, with a mortality rate of 22–32% [186]. Despite this, Gray et al. [185] still found that both single-strain and multi-strain probiotics significantly reduced the odds of NEC and death in preterm infants.

For a probiotic consisting of a total of five strains, FloraBaby is a common combination probiotic. This probiotic formulation typically consists of *B. breve*, *B. bifidum*, *B. infantis*, *B. longum*, and *L. rhamnosus* [83,187,188,189,190,191]. Numerous studies were conducted on preterm infants to find out if FloraBaby was beneficial for NEC prevention [83,187,188,189,190]. In terms of NEC and mortality, studies by Janvier et al. [83] and Singh et al. [188] were in agreement that FloraBaby, as a prophylactic probiotic, was feasible in NEC prevention in preterm infants. Janvier et al. [83] demonstrated that the use of probiotics—FloraBaby (2 × 10^9^ CFU/0.5 g) administered at the time of the first feed until the infant reached 34 weeks’ postmenstrual age—decreased the frequency of NEC in infants significantly from 9.8% to 5.4%, the rate of neonatal death reduced from 9.8% to 6.8% (non-significant), and the combined outcome of death or NEC decreased from 17% to 10.5% (significant), after probiotics administration. [83]. On the other hand, 79.1% infants in a study by Singh et al. [188] were administered with FloraBaby (2 × 10^9^ CFU/0.5 g), while 19.8% received a single-strain probiotic—Biogaia (10^8^ CFU/five drops). The period of probiotics began around the initiation of feeds and continued until 34 weeks postmenstrual age or the infant was transferred to a step-down unit. Overall, they found preterm infants fed probiotics showed a significant reduction in NEC, mortality, and composite outcome of NEC or mortality, but with no significant reduction in the LOS rate among survivors. The authors further conducted a post hoc analysis of infants receiving only the probiotic FloraBaby compared to those without prophylactic probiotics. The findings showed that FloraBaby was associated with a reduced NEC, mortality, and composite outcome of NEC or mortality. Furthermore, no significant differences were found in the rate of NEC requiring surgery, LOS, and parenteral nutrition days between the two groups. Sato et al. [187] also found preterm infants who were exclusively fed human milk along with daily FloraBaby (2 × 10^9^ CFU) probiotic had a significant reduction in NEC (5.2% vs. 1.1%). The probiotics were discontinued once infants achieved a corrected gestational age of 34 weeks. On an additional note, Alshaikh et al. [191] found that infants fed breast milk throughout the first 4 weeks of life who were on FloraBaby (*B. breve* HA-129 (1.2 billion CFU), *B. bifidum* HA-132 (800 million CFU), *B. infantis* HA-116 (600 million CFU), *B. longum* subsp. *longum* HA-135 (400 million CFU), *L. rhamnosus* (1.0 billion CFU)) had increased levels of fecal *Bifidobacterium* and sustained levels of *Lactobacillus*. Subjects were fed with probiotics until hospital discharge or 37 weeks corrected gestational age. The findings showed that within 2 weeks of feeding the probiotics, subjects became *Bifidobacterium*-dominant and continued to be dominant after probiotic cessation [191]. In terms of diversity, beta diversity analysis had a marked shift in bacterial community during and 2 weeks after stopping probiotics. Additionally, there were modifications to the intestinal mycobiome with a distinct anti-Candida effect [191]. Overall, these studies demonstrated that FloraBaby has potential benefits, including reduced NEC incidence, reduced mortality, and shifts in microbial dominance, alongside alterations in intestinal mycobiome with a distinct anti-Candida effect. However, not all studies reported consistent benefits of FloraBaby on NEC and mortality. In particular, Juber et al. [189] and Que et al. [190] found that FloraBaby (2 × 10^9^ CFU) did not affect the NEC rate/incidence or mortality.

Amini et al. [192] used a six-strain probiotic combination (*S. thermophilus*, *L. rhamnosus*, *L. acidophilus*, *L. bulgaricus*, *B. infantis*, *L. casei*) with an added fructo-oligosaccharide prebiotic and found that it had beneficial effects for the treatment and prevention of NEC. The incidence of NEC and C-reactive protein surge showed a significant difference between the case and control groups. Comparing the case given multi-strain powder probiotic infant formula and the control groups given enteral nutrition without probiotics, NEC grade I was 16.7% vs. 26.7; NEC grade II was 0% vs. 20%, while the increase in C-reactive protein was 6.7% vs. 30% [192]. The findings of NEC were relatively consistent with Fernandez-Carrocera et al. [193], who also used a combination probiotics of six different bacteria strains (*L. acidophilus* 1.0 × 10^9^ CFU/g, *L. rhamnosus* 4.4 × 10^8^ CFU/g, *L. casei* 1.0 × 10^9^ CFU/g, *L. plantarum* 1.76 × 10^8^ CFU/g, *B. infantis* 2.76 × 10^7^ CFU/g, *S. thermophilus* 6.6 × 10^5^ CFU/g, per pack), agreeing that the probiotics had the potential to reduce NEC risk. Although the study found that the probiotics did not contribute to NEC risk reduction, it did show a decrease in NEC frequency in the probiotic group (8%) vs. the control group (16%). Additionally, when the combined risk of NEC or death was calculated as a post hoc analysis, there was a significantly lower risk for the probiotic group. There were also findings on combination probiotics being beneficial against LOS, with Sinha et al. [194] indicating that microbial interference as a result of probiotic supplementation aided in reducing infant morbidity, as observed by a non-significant 21% reduction in sepsis risk and a 15 day delay in sepsis onset in infants fed with probiotics (*S. thermophilus*, *B. breve*, *B. longum*, *B. infantis*, *L. acidophilus*, *L. plantarum*, *Lactobacillus paracasei* (reclassified as *Lacticaseibacillus paracasei* [129])), and *Lactobacillus delbrueckii* subsp. *bulgaricus* for 30 days (Table 3).

**Table 3:** The use of multi-strain probiotics in preterm infants, focusing on its effect on necrotizing enterocolitis (NEC), sepsis, and mortality.

Probiotic (Number of Strains)	Prebiotic	Dosage/Type of Feeding	Probiotic Treatment Period	Sample Size, Gestational Age (GA^b^), Birth Weight (BW^c^)	Country	NEC/LOS Findings	Study
*Lactobacillus acidophilus*, *Lactobacillus bulgaricus*, *Bifidobacterium bifidum* (3)	Fructo-oligosaccharide.	Each capsule had 500 mg blend: *Lactobacillus acidophilus* 2 billion CFU^a^; *Lactobacillus bulgaricus* 1 billion CFU; *Bifidobacterium bifidum* 1 billion CFU; and fructo-oligosaccharide 100 mg. A total of 0.5 mL (3 × 10^9^ CFU) once daily. Mother’s milk.	Started from first feeding by dropper or tube until discharged.	Sample size: *n* = 119 (probiotics: *n* = 59; placebo: *n* = 60) GA ≤ 35 completed weeks BW ≤ 2000 g	Bangladesh	NEC and mortality: Significantly lower NEC incidence (1.7% vs. 13.3%) and mortality compared to the placebo group.	[178]
*Bifidobacterium lactis* (BB-12^®^), *Bifidobacterium infantis* (BB-02™), *Streptococcus thermophilus* (TH-4^®^) (3) (Similac^®^ Probiotic Triblend) (Abbott, Abbott Park, IL, USA; manufactured by Chr. Hansen, HØrsholm, Denmark)	Not available	One billion CFU of *Bifidobacterium lactis* (BB-12^®^), *Bifidobacterium infantis* (BB-02™), and *Streptococcus thermophilus* (TH-4^®^) mixed in 3 mL of sterile water. Once daily. All infants started on human milk; 50% of infants discharged on mother’s milk.	First dose of probiotic administered. within 48 h of birth and after first feed of mother’s colostrum (or donor breast milk if colostrum was not available). Probiotic ceased when infant reached 35 weeks postmenstrual age or was discharged.	Sample size: *n* = (probiotics: *n* = 125; no probiotics: *n* = 126) GA < 32 weeks BW < 1500 g	USA	NEC, LOS (late-onset sepsis), and mortality: Decreased incidence of NEC from 6.3% (no probiotic) to 1.6% (probiotic group), but the differences in NEC, LOS, and death were not significant after adjusting for multiple variables. No adverse effects.	[179]
*Bifidobacterium infantis* (BB–02), *Streptococcus thermophilus* (TH–4), *Bifidobacterium lactis* (BB-12) (3) (ABC Dophilus Probiotic Powder for Infants, Solgar^®^, Leonia, NJ, USA)	Not available	*Bifidobacterium infantis* (BB–02 300 × 10^6^), *Streptococcus thermophilus* (TH–4 350 × 10^6^), and *Bifidobacterium lactis* (BB-12 350 × 10^6^) with 1 × 10^9^ total organisms per 1.5 g in a maltodextrin base powder. Administered daily by reconstituting with breast milk or formula.	Given only when an infant received at least 1 mL of milk every 4 h. The intervention was withheld during periods when infants were nil orally until discharged from hospital or reached corrected age.	Sample size: *n* = 1099 (probiotics: *n* = 548; placebo: *n* = 551) GA < 32 weeks BW < 1500 g	Australia and New Zealand	NEC: Reduced NEC of Bell’s stage II or more significantly (2.0% versus 4.4%). LOS and mortality: No significant difference in definite LOS or all-cause mortality.	[180]
*Lactobacillus acidophilus* (subsp. *Lactobacillus gasseri*) PTA-5845, *Bifidobacterium infantis* PTA-5843, *Enterococcus faecium* PTA 5844 (3) (Linex^®^ capsule, produced by Lek d.d., Ljubljana, Slovenia)	Not available	Per capsule contained at least 1.2 × 10^7^ CFU *Lactobacillus acidophilus* (subsp. *Lactobacillus Gasseri*) PTA-5845, *Bifidobacterium infantis* PTA-5843, and *Enterococcus faecium* PTA 5844 in the ratio of 1.5:1:1.5. A total of 0.6 × 10^7^ CFU given twice daily with the first portions of milk (breast milk/milk formula) until discharge.	Administered with the first portions of milk until discharge.	Sample size: *n* = 80 (probiotics: *n* = 40; no probiotics: *n* = 40) GA < 33 weeks BW < 1500 g	Slovenia	LOS: Prevented late-onset infections. Reduced LOS frequency (significant). No side effects.	[181]
*Streptococcus thermophilus*, *Bifidobacterium infantis*, *Bifidobacterium bifidum* (3) (ABC Dophilus) (Solgar^®^, Leonia, NJ, USA)*	Not available	VLBW^d^ 1000–1500 g: 1.05 × 10^9^ CFU^a^ s/day. Standard mix of ½ tsp in 3 mL mother’s breast milk/formula and given once daily. ELBW^e^ < 1000 g: 0.5 × 10^9^ CFUs/day. If 1 mL is maximum oral/oral gastric volume allowable, mix ¼ tsp in 3 mL sterile water and give 1 mL three times daily.	Started from first day of oral/orogastric feeds until corrected GA of 36 weeks or discharge home or to another facility. Average duration of probiotics is around 37 days.	Sample size: *n* = 580 (probiotics: *n* = 291; control (no probiotics): *n* = 289) GA < 33 weeks BW < 1500 g	USA	NEC: Safe for preventing NEC in VLBW infants. Study (observational) was unable to detect changes in outcome due to lower incidence of NEC (2.8%) and NEC scare (stage I NEC) (2.8%).	[182]
*Lactobacillus rhamnosus*, *Lactobacillus casei*, *Lactobacillus plantarum*, *Bifidobacterium animalis* (4)	Fructo-oligosaccharides and galacto-oligosaccharides.	*Lactobacillus rhamnosus* (4.1 × 10^8^ CFU) + *Lactobacillus casei* (8.2 × 10^8^ CFU) + *Lactobacillus plantarum* (4.1 × 10^8^ CFU) + *Bifidobacterium animalis* (4.1 × 10^8^ CFU) together with 383 mg of fructo-oligosaccharides and 100 mg of galacto-oligosaccharides as prebiotic content. 2 × 1 sachet once enteral nutrition reached 50–60 mL/kg. Mother’s milk/formula milk/mixed (combination of mother’s milk and formula milk).	Started when the amount of the diet in one meal time exceeded 2 mL, and when the enteral nutrition reached 50–60 mL/kg, it was set as 2 × 1 sachet until discharge. Targeted probiotics to be administered 4–6 weeks. Start time of the probiotics ranged between postnatal second and seventh days, with a mean of 4.3 ± 1.5 days. Infants had a mean 36.5 ± 12.6 days of probiotics, reaching the total dose in a mean 9.8 ± 3.4 days.	Sample size: *n* = 110 (probiotics: *n* = 70; no probiotics: *n* = 40) GA ≤ 32 weeks BW ≤ 1500 g	Turkey	NEC and mortality: Significantly lower NEC incidence and mortality rates in the probiotic group.	[183]
*Lactobacillus acidophilus*, *Bifidobacterium longum*, *Bifidobacterium bifidum*, *Bifidobacterium lactis* (4) (Prowel (Batch PWS3002C) by Alkem, Mumbai*, Maharashtra*, India*)	Not available	*Lactobacillus acidophilus* 1.25 billion, *Bifidobacterium longum* 0.125 billion, *Bifidobacterium bifidum* 0.125 billion, and *Bifidobacterium lactis* 1.0 billion per 1 g sachet. For VLBW neonates, the dose of 6 × 10^9^ CFU/day of lactobacillus, as half of 1 g sachet. For ELBW and <32 weeks, the starting dose should be 1.5 × 10^9^ CFU/day until they reached enteral feeds of 50–60 mL/kg/day, then the dose was increased to 3 × 10^9^ CFU/day. Administered twice daily with breast milk.	Probiotics administered from the first 72 h for 6 weeks or until discharged.	Sample size: *n* = 112 (probiotics: *n* = 56; placebo: *n* = 56) GA < 37 weeks BW < 2500 g	India	Sepsis: Decreased invasive fungal (*Candida*) sepsis. Occurrence of LOS (inclusive of fungal sepsis) was 55.4% in the probiotics group and 75% in the placebo group. Decreased enteral fungal (*Candida*) colonization.	[184]
**Four different types of known probiotics:** (i) *Lactobacillus* (71%) (1), (ii) Ultimate Flora (27%) (RenewLife^®^, Sunrise, FL, USA)*: (*Bifidobacterium* and *Lactobacillus* species) (2) (iii) ABC Dophilus (6%) (Solgar^®^, Leonia, NJ, USA)*: (*Bifidobacterium*, *Lactobacillus*, *Streptococcus* species) (3) (iv) Align (0.4%) (Procter & Gamble^®^ (P&G), Cincinnati, OH, USA)*: (*Bifidobacterium*) (1) (v) Others (3%)	Not available	Not available	Received probiotics during the first 120 postnatal days. The median start day of probiotics was 4 and the median duration of exposure was 50 days.	Sample size: *n* = 35,985 (probiotics: *n* = 2178; no probiotics: *n* = 33, 807) GA (median): 28 weeks and <120 postnatal days BW (median): 1020 g	USA and Puerto Rico	NEC and mortality: Conditional regression analysis showed a significant reduction in odds of NEC and death in the probiotic group. Sepsis: Higher odds for Candida infections in the probiotic group.	[185]
FloraBABY (Renew Life^®^ Canada, Oakville, Ontario, Canada): *Bifidobacterium breve*, *Bifidobacterium bifidum*, *Bifidobacterium infantis*, *Bifidobacterium longum*, *Lactobacillus rhamnosus* GG (5)	Not available	*Bifidobacterium breve*, *Bifidobacterium bifidum*, *Bifidobacterium infantis*, *Bifidobacterium longum*, and *Lactobacillus rhamnosus* GG (2 × 10^9^ CFU/0.5 g). Mixed with 1 mL water just before milk (breast milk/formula) feeding once daily and continued until the infant reached 34 weeks postmenstrual age.	Started at the time of the first feed and continued until the infant reached 34 weeks postmenstrual age. Infants who developed NEC while receiving probiotics had the probiotics discontinued for the period of being nil by mouth and later restarted. Average duration of therapy: 24 days; average day of starting probiotics: day 4.3.	Sample size: *n* = 611 (probiotics: *n* = 294; pre-probiotic: *n* = 317) GA < 32 weeks BW (mean): 1220 g (probiotic); 1207 g (pre-probiotic)	Canada	NEC: Decreased frequency of NEC significantly (from 9.8% to 5.4%). Mortality: Decreased rate of neonatal death non-significantly (from 9.8% to 6.8%). NEC or mortality: Decreased in the combined outcome of death or NEC significantly (from 17% to 10.5%).	[83]
Florababy (Renew Life^®^ Canada)(given to 79.1% infants): *Bifidobacterium breve*, *Bifidobacterium bifidum*, *Bifidobacterium infantis*, *Bifidobacterium longum* and *Lactobacillus rhamnosus* GG (5) Single-strain Biogaia (Ferring Inc.) (BioGaia^®^ AB, Stockholm, Sweden)* (given to 19.8% infants): *Lactobacillus reuteri* (1)	Not available	*Bifidobacterium breve*, *Bifidobacterium bifidum*, *Bifidobacterium infantis*, *Bifidobacterium longum*, and *Lactobacillus rhamnosus* GG (2 × 10^9^ CFU/0.5 g). *Lactobacillus reuteri* (10^8^ CFU/five drops).	Administration began around initiation of feeds and continued until 34 weeks postmenstrual age/infant transferred to a step-down unit.	Sample size: *n* = 3093 (probiotics: *n* = 652; no probiotics: *n* = 2441) GA < 29 weeks BW (mean): 924 g (probiotic); 920 g (no probiotic)	Canada	NEC or mortality: Significantly lower rates of mortality and the composite outcome of NEC or mortality in the probiotic groups. LOS: Significantly higher LOS among survivors fed probiotics. Post hoc analysis of neonates receiving Florababy: Reduction in NEC, mortality, and composite outcome of NEC or mortality. No significant difference in the rate of NEC requiring surgery, LOS, and parenteral nutrition days between the FloraBaby probiotic group and the no probiotics group.	[188]
Florababy (RenewLife^®^ Canada, Brampton, Ontario, Canada): *Bifidobacterium* and *Lactobacillus* species (5)	Not available	500 mg daily (2 × 10^9^ CFU) *Bifidobacterium breve*, *Bifidobacterium bifidum*, *Bifidobacterium infantis*, *Bifidobacterium longum*, and *L. rhamnosus*. Exclusively human milk then human milk added with human milk-derived fortifier.	Started when enteral nutrition commenced. Probiotics and human milk–derived fortifier were discontinued once infants achieved a corrected GA of 34 weeks.	Sample size: *n* = 399 (post-intervention: 265; pre-intervention: 134) GA: probiotics: 29.5 ± 2.4 weeks; no probiotics: 29.6 ± 2.1 weeks BW: 1000 –1499 g	USA	NEC: Significant NEC reduction (5.2% vs. 1.1%) in infants fed exclusively human-milk and with daily probiotic supplement.	[187]
Florababy (Ultimate Flora Baby Probiotic^®^, RenewLife^®^, Sunrise*, Florida*): *Bifidobacterium breve*, *Bifidobacterium bifidum*, *Bifidobacterium infantis*, *Bifidobacterium longum*, *Lactobacillus rhamnosus* GG (5)	Not available	2 × 10^9^ CFU^a^/0.5 g. Once daily (0.5 g of probiotic to 2.5 mL of 2.5% dextrose in sterile water) via enteral gavage before enteral feed. Exclusively breast milk, exclusively formula, or mixed (combination of breast milk and formula milk).	Probiotics administered to infants who were at least 3 days old, born at <33 0/7 weeks GA, with a corrected post-menstrual age of at least 24 0/7 weeks, who were also receiving intake of at least 6 mL of enteral feedings per day. Probiotics continued daily until infant reaches corrected postmenstrual age of 36 0/7 weeks.	Sample size: *n* = 37 (post-probiotics: *n* = 23; pre-probiotics: *n* = 14) GA: post-probiotics: 28.7 (±1.1) weeks; pre-probiotics: 30.6 (±1.2) weeks BW: post-probiotics: 1332 (±216) g; pre-probiotics: 1628 (±240) g	USA	NEC and mortality: No significant differences in rates of modified Bell’s stage ≥ IIa NEC (pre-probiotic 2.1% vs. post-probiotic 1.5%) or all-cause pre-discharge mortality (pre-probiotic 8.4% vs. post-probiotic 7.4%) in VLBW infants. No impact on NEC incidence on ELBW infants (pre-probiotics 1.6% vs. post-probiotics 4.1%).	[189]
Florababy (RenewLife^®^, Sunrise, FL, USA)*: *Bifidobacterium breve*, *Bifidobacterium bifidum*, *Bifidobacterium infantis*, *Bifidobacterium longum*, and *Lactobacillus rhamnosus* (5)	Not available	*Bifidobacterium breve*, *Bifidobacterium bifidum*, *Bifidobacterium infantis*, *Bifidobacterium longum*, and *Lactobacillus rhamnosus* (2 × 10^9^ CFU). A total of 0.5 g of probiotics mixed with 1 mL of sterile water once daily. Breast milk and/or human milk fortifier.	Started from the time of initiation of feeds until 35 weeks corrected GA. Probiotics discontinued for any infant who developed necrotizing enterocolitis.	Sample size: *n* = 665 (probiotics: *n* = 310; no probiotics: 355) GA (median), weeks: 28.6 (probiotic); 28.4 (no probiotic) BW: VLBW infants (median): 1060 g (probiotic); 1090 g (no probiotic)	Canada	NEC: No significant impact on incidence of NEC (Bell’s stage II or III), or mortality.	[190]
Protexin-Restore (Probiotic International Ltd.^+^, United Kingdom): *Streptococcus thermophilus*, *Lactobacillus rhamnosus*, *Lactobacillus acidophilus*, *Lactobacillus bulgaricus*, *Bifidobacterium infantis*, *Lactobacillus casei* (6)	Fructo-oligosaccharide.	A total of 1 billion bacteria (*Streptococcus thermophilus*, *Lactobacillus rhamnosus*, *Lactobacillus acidophilus*, *Lactobacillus bulgaricus*, *Bifidobacterium infantis*, *Lactobacillus casei*) and 990 mg fructo-oligosaccharide as prebiotic. In the first 10 days of life, 1 g probiotic infant formula administered with enteral feeding in 8 to 10 divided doses between two breast milk feedings for ≥7 days.	Probiotic started after vital signs were stable. Probiotic administered for ≥13 days.	Sample size: *n* = 60 (probiotics: *n* = 30; no probiotics: *n* = 30) GA: <32 weeks BW: 750 g–1500 g	Iran	NEC: Significant difference in NEC incidence (NEC grade 1: 16.7% vs. 26.7%) (NEC grade 2: 0% vs. 20%) and C-reactive protein (6.7% vs. 30%). Positive effects in NEC prevention and treatment, especially NEC grade III in ELBW and VLBW neonates.	[192]
*Lactobacillus acidophilus*, *Lactobacillus rhamnosus*, *Lactobacillus casei*, *Lactobacillus plantarum*, *Bifidobacteruim infantis*, *Streptococcus thermophilus* (6) (Laboratorio Italmex SA, Mexico City, Mexico)	Not available	*Lactobacillus acidophilus* 1.0 × 10^9^ CFU/g, *Lactobacillus rhamnosus* 4.4 × 10^8^ CFU/g, *Lactobacillus casei* 1.0 × 10^9^ CFU/g, *Lactobacillus plantarum* 1.76 × 10^8^ CFU/g, *Bifidobacteruim infantis* 2.76 × 10^7^ CFU/g, and *Streptococcus thermophillus* 6.6 × 10^5^ CFU/g per pack. Fed 1 g probiotic per day diluted in mother’s milk/formula.	No clear information.	Sample size: *n* = 150 (probiotics: *n* = 75; placebo: *n* = 75) GA (median): probiotic: 31.2 (26–35.4); placebo: 31 (27–36) (preterm) BW < 1500 g	Mexico	NEC: Promising strategy in NEC risk reduction. Reduction in NEC frequency (8% vs. 16%). Post hoc analysis: The combined risk of NEC or death had a significantly lower risk for the probiotic group.	[193]
VSL#3 (Prepared by CD Pharma India Pvt. Ltd., Delhi*, India*): *Streptococcus thermophilus*, *Bifidobacterium breve*, *Bifidobacterium longum*, *Bifidobacterium* *infantis*, *Lactobacillus acidophilus*, *Lactobacillus plantarum*, *Lactobacillus paracasei* and *Lactobacillus delbrueckii* subsp. *bulgaricus*) (8)	Not available	Administered at a dose of 10 billion CFU. Mixed in expressed breast milk.	Administered from the third day of life for 30 days and followed up for 2 months.	Sample size: *n* = 1340 (probiotics: *n* = 668; placebo: *n* = 672) GA: Not available (low-birth weight infants) BW: 1500 g–2500 g	India	Sepsis: Reduced risk of neonatal sepsis (non-significant 21%). A total of 15 days delay in the onset of sepsis in the probiotic group.	[194]

^+^ The company Probiotic International Ltd. has been changed to ADM Protexin Ltd.; * The information on countries for probiotics products was obtained through respective official websites; ^a^ Colony-forming unit; ^b^ Gestational age; ^c^ Birth weight; ^d^ Very low birth weight; ^e^ Extremely low birth weight.

### 4.3. Single vs. Multiple Strain Probiotics

Some studies suggested that multi-strain probiotics were more effective than single-strain probiotics. Uberos et al. [195] studied both single-strain probiotic LGG (ATCC 53103) (10^9^ CFU) and dual-strain Infloran (10^9^ CFU *L. acidophilus* (ATCC 4356) and 10^9^ CFU *B. bifidum* (ATCC 15696)), and concluded that probiotics were linked with reduced NEC, LOS, and mortality in preterm infants. However, they found that in the subgroup analysis, supplementation with the combination probiotic Infloran demonstrated fewer LOS complications than single probiotic LGG. Therefore, this suggests that combination probiotics is the preferred approach, while also emphasizing that strain selection is highly important [195], which was in agreement with Beck et al. [94], as the choice of probiotics could affect gut microbiome development, and it is important to understand the short-term and long-term impacts of probiotics at the strain level. In terms of the effect on probiotics and the gut microbiome, a study conducted in France by Hays et al. [196] found that upon administering the single-strain probiotic *B. lactis* alone, *B. longum* alone, and dual-strain probiotic *B. lactis* and *B. longum* for 3 weeks, the microbiological family *Bifidobacterium* spp. were detected more often in infants who received *B. lactis* either alone, followed by combination with *B. longum*, and lastly, *B. longum* alone. This suggests that in the case of *B. longum*, the addition of *B. lactis* may have contributed to the biological activity, resulting in the dual-strain probiotic being better at surviving the gut passage.

Nonetheless, there are studies that showed no increased beneficial effect of using more than one probiotic strain. In terms of NEC, four studies analyzed in this review were in agreement that the use of combination probiotics did not have a better outcome than the use of a single probiotic. Meyer et al. [197] found no significant difference in NEC reduction between the use of the single-strain probiotic LGG (ATCC 53103) (6 × 10^9^ CFU) added with the prebiotic bovine lactoferrin versus the combination probiotic Infloran (10^9^ CFU each of *L. acidophilus* (ATCC 4356) and *B. bifidum* (ATCC 15696)). Additionally, Gomez-Rodriguez et al. [198] found no differences in NEC incidence between preterm infants fed single-strain probiotic *Lactobacillus acidophilus boucardii* and multi-species probiotic combination containing *L. acidophilus*, *L. rhamnosus*, *L. casei*, *L. plantarum*, *B. infantis*, and *S. thermophilus*. The finding was consistent with Priyadarshi et al. [199], who found no differences in the clinical efficacy between single- (*B. breve* M-16 V) and dual-strain (Infloran) probiotic prophylaxis for preventing NEC in preterm infants. Furthermore, a recent paper published in 2023 by Korcek et al. [200] also found no difference in the rate of NEC (3.5% vs. 2.6%), LOS (15.4% vs. 12.3%), and mortality (0.9% vs. 1.8%) between groups of preterm infants fed multi-species probiotics (consisting of *L. rhamnosus* 45%, *L. casei* 15%, *L. acidophilus* 15%, *B. infantis* 15%, *B. bifidum* 10% along with prebiotics fructo-oligosaccharides) or single-species probiotics (consisting of *B. breve* BR03 and B632).

Based on the evidence, single-strain and muti-strain probiotics show comparable efficacy. However, further research considering probiotic strains and study population is needed to determine whether combination probiotics consistently provide better health outcomes than single-strain probiotics.

## 5. Future Direction for Improvement

Over the years, extensive clinical research has investigated the use of probiotics in preterm infants. Despite substantial evidence showing that probiotics have potential beneficial effects in preterm infants, many studies remain heterogenous or exploratory [201,202,203,204]. As a result, the clinical adoption of probiotics as a standard supplement or treatment protocol in the NICU for preterm infants is not yet a globally accepted standard of care. Some major concerns limiting its adoption as a standard clinical protocol for preterm infants include its safety and long-term outcomes, as many studies discontinue administrating probiotics at the time of discharge [205,206].

Regarding safety, we agree with Van Den Akker et al. [207] that the careful adoption of probiotics, guided by evidence and practicality, can maximize their life-saving potential without compromising safety and efficacy. Despite the reported potential benefits of probiotic supplementation in preterm infants, safety must be a priority consideration in this vulnerable population, as they possess immature immune systems and compromised intestinal barrier functions [208]. Some concerns regarding the administration of live microorganisms include probiotic-associated sepsis, with case reports and systematic reviews documenting the isolation of administered strains from blood cultures, implying translocation from the gut into systemic circulation in rare cases [209,210]. Meta-analyses have reported low rates of serious infections directly attributable to administered probiotic organisms, suggesting an overall favorable safety profile in preterm infants [211]. For example, a meta-analysis of over 20,323 probiotic-exposed infants identified probiotic sepsis in only eight cases [211]. In rare cases of probiotic-associated sepsis, advanced molecular methods such as whole genome sequencing and 16S rDNA sequencing are necessary to confirm that the strain isolated from blood matches the administered probiotic, as traditional identification methods such as Vitek 2 system may yield incorrect identifications [209]. Nevertheless, these rare reports of probiotic-associated sepsis underscore that adverse events, while uncommon, are possible—especially in extremely low birth weight infants and when product contamination or misidentification happens [44,209,211]. Taken together, these findings highlight the importance of rigorous strain identification, strict manufacturing quality, and careful monitoring of adverse effects in both research and clinical practices [208,210,212]. Given the limited long-term safety data available for preterm infants, future studies should include systematic safety assessments alongside efficacy outcomes when considering probiotic use in this vulnerable population to ensure that its use confers a net benefit without unintended harm [208].

Despite growing evidence on the short-term benefits of early probiotic intervention, its long-term consequences remain unclear, particularly whether it induces sustained ‘programming’ of the microbiota and immune system into adulthood or whether these effects are transient. For example, studies in preterm infants have shown that probiotic species and their modulatory effects often do not persist once supplementation is stopped, suggesting a potential reversion to baseline microbiota [205,213]. Although early gut colonization plays a recognized role in shaping immune development and may influence long-term health outcomes [214,215,216], there is still limited evidence demonstrating that transient probiotic exposure results in durable microbiota or immune programming. This is partly due to the lack of longitudinal and mechanistic studies assessing whether early microbial modulation leads to sustained molecular and immunological changes. Future longitudinal human studies are therefore needed to clarify the persistence and functional significance of these effects.

It is worth noting that all the included studies (Table 1, Table 2 and Table 3, and Section 4.3) relied exclusively on blood culture-positive or clinical sepsis for sepsis, and clinical/radiological criteria for NEC—none employed polymerase chain reaction (PCR)-based or molecular diagnostic. While this uniformity reduces methodological heterogeneity from culture versus molecular methods, it means the current evidence applies specifically to culture-positive and clinically diagnosed sepsis. Future trials incorporating molecular methods as an adjunct to culture would clarify whether probiotics also benefit infants with culture-negative sepsis that is detectable only by PCR.

More research is needed, as the current evidence on the use of probiotics in preterm infants remains incomplete. For instance, our analysis showed that several studies had a limited focus on factors that could affect probiotic efficacy, such as the type of feeding [169,217]; antibiotic exposure [218]; timing of probiotic administration (initiation of first dose) [139,218,219]; exact duration of probiotic administration [220]; and baseline microbiome [218,221]. Most importantly, probiotics are strain-specific and disease-specific, making it crucial for studies to distinguish between strains of the same species [222,223], as different species of probiotic strains may have varying properties and physiological functions. Furthermore, probiotics are not a homogeneous intervention, warranting a separate analysis for each probiotic intervention [188,224]. Hence, all the factors mentioned must be taken into consideration when designing a trial and interpreting outcomes, so that results from these trials would be more reliable with better reproducibility and could more likely be generalized for the clinical application of probiotics in preterm infants.

## 6. Conclusions

NEC and LOS are devastating diseases affecting preterm infants that are both associated with gut dysbiosis. As a result of the factors around the delivery of preterm infants, as well as the therapeutic interventions required, these infants are highly unlikely to spontaneously develop the appropriate balance of beneficial gut bacteria that would have then assisted with reducing the growth of pathogenic organisms and aided with gut immunity and maturation. In recent years, probiotics have been widely studied as a possible method to overcome this and are slowly being accepted internationally by neonatologists and pediatricians. Numerous studies on probiotics as a supplement have been conducted to investigate their effects in preventing and/or treating NEC and sepsis.

Overall, studies suggest that probiotic supplementation may reduce the risk of NEC, sepsis, and mortality in preterm infant populations. The majority of studies included in this review focused on the effects of probiotics on NEC, while sepsis and mortality were evaluated less frequently. Based on the studies we reviewed, *Bifidobacterium* and *Lactobacillus*, alone or in combination, appear to be the more commonly used probiotic species in preterm infant studies. Among the studies using combination probiotics, Infloran (*L. acidophilus* and *Bifidobacterium* spp. (either *B. bifidum* or *B. infantis*)) and Florababy (*B. breve*, *B. bifidum*, *B. infantis*, *B. longum*, and *L. rhamnosus*) are among the most commonly investigated multi-strain probiotics used in preterm infant studies. Although the findings were heterogeneous, approximately half of the included studies indicated that probiotic supplementation is generally associated with positive outcomes on NEC and/or LOS and/or mortality in preterm infants. With that being said, the variability in outcome definitions (NEC alone, sepsis alone, mortality, or composite endpoints such as NEC or sepsis or death) and analytical methods (univariate vs. adjusted models, conditional logistic regression, or post hoc analyses) likely contributes to the heterogeneity and limits direct comparisons between studies. Moreover, no single or combination of probiotic species consistently outperformed the other in terms of NEC and sepsis outcomes, and the findings were inconsistent despite administering the same probiotic species across different studies. Therefore, despite the promising potential of probiotics, the certainty of these benefits and their magnitude may vary depending on the study design and population characteristics.

In summary, probiotics represent a promising area of neonatal care, though continued investigation will be key to refining probiotic strategies and unlocking their full benefits for this vulnerable population. More standardized and high-quality research is necessary to establish consistent guidelines for probiotic use in preterm infants. A higher focus should be placed on investigating the types/strains of probiotics; their properties; the optimum dosage; the best time to initiate administration; the duration of treatment; and whether the type of feeding plays a part in probiotics’ efficacy, as well as its mechanisms of action, in order to provide positive health outcomes in terms of NEC and LOS in preterm infants.

## Data Availability

No new data were created or analyzed in this study. Data sharing is not applicable.
